# Disrupting Biofilm Tolerance by Ionic Microbubble-Mediated Copper Ion Surge for Infection Clearance

**DOI:** 10.1021/acsnano.5c08035

**Published:** 2025-07-31

**Authors:** Xiaoye Li, Qiang Li, Ao He, Meng Dang, Yu Zhang, Minjin Wang, Qinhong Sun, Zhuo Dai, Meng Ding, Jingben Zheng, Yongbin Mou, Weijun Xiu, Heng Dong

**Affiliations:** † Nanjing Stomatological Hospital, Affiliated Hospital of Medical School, Institute of Stomatology, 12581 Nanjing University, 30 Zhongyang Road, Nanjing 210008, China; ‡ Department of Thoracic Surgery, Jiangsu Key Laboratory of Molecular and Translational Cancer Research, 12461 Jiangsu Cancer Hospital & Jiangsu Institute of Cancer Research & Nanjing Medical University Affiliated Cancer Hospital, Nanjing 210009, China; § Department of Biomedical Engineering (BME), 37580 National University of Singapore, 15 Kent Ridge Cres, Singapore 119276, Singapore

**Keywords:** biofilm tolerance, microbubble, cuproptosis-like death, immune, intracellular bacterial clearance

## Abstract

Bacterial infections caused by drug-resistant bacteria persist due to biofilm-mediated tolerance, which limits the efficacy of both antimicrobial agents and host immune defenses. Here, we develop ionic microbubbles (MB-CuTA) self-assembled by Fe_3_O_4_@CuTA nanoparticles to enhance copper ion-mediated antibiofilm therapy. Upon ultrasound activation, MB-CuTA undergoes inertial cavitation, disrupting biofilm integrity and generating a localized surge of copper ions. This process achieves a dual therapeutic effect: (1) disruption of bacterial metabolic homeostasis, thereby overcoming the intrinsic resistance of biofilms to conventional antimicrobial agents, and (2) activation of cellular immunity to effectively counteract bacterial immune evasion mechanisms. By breaking biofilm tolerance through both metabolic and immunological pathways, our strategy enables deep copper ion penetration in biofilms and effective infection clearance in both mouse implant infection and peritonitis infection models. Our approach introduces a biofilm tolerance disruption method through inducing bacterial cuproptosis-like death and cellular immunity activation, offering a promising strategy against biofilm infections.

## Graphical abstract

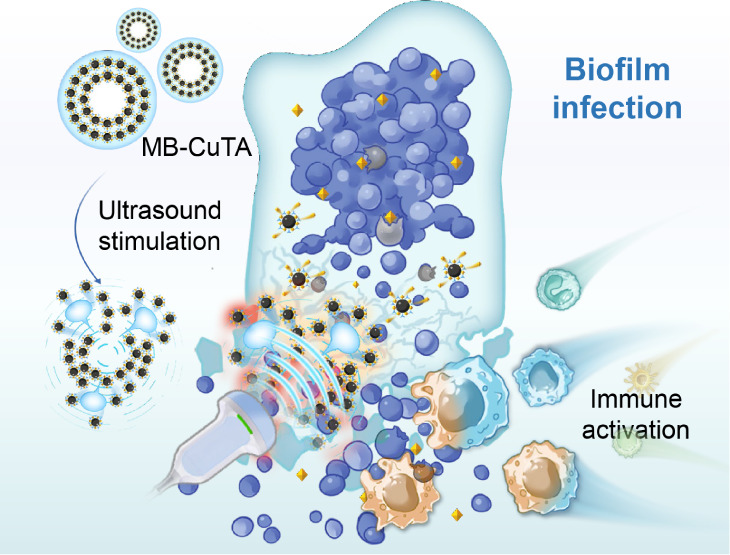

## 1 Introduction

Biofilm-associated infections present a significant challenge to modern medicine due to their resilience against conventional antimicrobial treatments and their ability to evade host immune responses.^1–4^ Biofilms are structured communities of bacteria encapsulated within an extracellular polymeric substance (EPS), which forms a protective matrix that fosters the persistence of bacteria.^5,6^ The biofilms exhibit remarkable tolerance to both antimicrobial agents and host immune responses, thereby rendering these infections particularly recalcitrant to conventional treatment.^2^ On one hand, the encapsulation of EPS around biofilms can inhibit the interaction of embedded bacterial cells with antimicrobials by impairing antimicrobial penetration and neutralizing or suppressing their activity, thereby creating a homeostatic microenvironment that protects internal bacteria for the attack of antimicrobial agents.^7,8^ On the other hand, biofilms establish a finely tuned tolerance homeostasis to evade the immune system by inhibiting cellular immunity and modulating adaptive immunity. They suppress phagocytosis through the EPS, induce immune cell apoptosis, and interfere with inflammatory signaling to suppress cellular immunity.^9^ Even when phagocytosed, some bacteria survive inside immune cells by resisting intracellular killing, leading to persistent infections and recurrent outbreaks.^10–12^ Additionally, biofilms promote Th2 cell differentiation, impair antigen presentation, and dysregulate B cell responses for inhibiting cellular immunity, leading to chronic infections and immune tolerance. These intricate defense mechanisms underscore the urgent need for innovative strategies to disrupt biofilm tolerance homeostasis and overcome its inherent tolerance mechanisms.

The equilibrium between antimicrobial resistance and immune modulation enables biofilms to maintain a resilient state, rendering them particularly difficult to eradicate by using conventional therapies. Metal ions, which are integral to biofilm stability, support microbial metabolic processes and bolster defense mechanisms.^11^ Recent research has underscored their potential as both immunomodulatory and antimicrobial agents, thereby opening up new avenues for therapeutic intervention.^13^ Among these, copper ions are especially noteworthy for their potent antimicrobial properties, including disruption of bacterial cell walls, induction of oxidative stress, and inhibition of essential metabolic pathways.^14–18^ However, the biofilm matrix presents a formidable barrier, often preventing copper ions from penetrating deeply enough to achieve effective inhibitory concentrations within the biofilm.^19^ Beyond their direct bactericidal effects, copper ions play a crucial role in immune modulation. They have been shown to promote macrophage polarization, activate dendritic cells (DCs), and enhance T cell responses, thereby boosting cell-mediated immunity.^20–23^ This dual functionality suggests that copper ions could effectively counteract biofilm-induced tolerance by disrupting immune evasion strategies and enhancing the bacterial clearance. Despite these promising implications, there is a notable lack of studies explicitly investigating the ability of copper ions to reverse biofilm-mediated immune modulation and restore cellular immune responses.^24^ Collectively, copper ion-based antibiofilm strategies hold significant potential to synergistically leverage both antimicrobial and immunomodulatory effects, thereby disrupting biofilm tolerance mechanisms. Through this dual-action approach, such strategies may ultimately enable a more effective treatment of biofilm-associated infections.

In this study, we propose an ionic microbubble system (MB-CuTA), constructed using copper-tannic-acid-coated Fe_3_O_4_ nanoparticles (Fe-CuTA), for the treatment of bacterial biofilm infections. Upon ultrasound (US) stimulation, MB-CuTA undergoes inertial cavitation, which effectively disrupts the biofilm architecture and promotes deep penetration of Fe-CuTA nanoparticles within the biofilm matrix. The released Fe-CuTA not only catalyzes the production of ROS for degradation of the biofilm structure, but also delivers high concentrations of copper ions. These copper ions disrupt bacterial metabolic homeostasis and induce cuproptosis-like cell death, resulting in potent bactericidal activity. Moreover, the sonoporation effect generated by microbubble cavitation significantly enhances macrophage uptake of Fe-CuTA nanoparticles, leading to increased intracellular copper ion accumulation and efficient eradication of phagocytosed bacteria.^25^ The released copper ions further potentiate immune activation by promoting M1 macrophage polarization, Th1 cell differentiation, N1-polarized neutrophil activation, and DC maturation. Together, these synergistic immunomodulatory effects foster a robust antimicrobial microenvironment, substantially improving bacterial clearance through enhanced cellular immune responses (Figure 1). Using a titanium implant Methicillin-resistant *Staphylococcus aureus* (MRSA) biofilm infection mouse model, we demonstrated that this strategy effectively disrupts biofilms, achieves a high bacterial killing efficiency (99.91%), and activates macrophage-mediated antimicrobial responses. In a peritonitis mouse model, the approach mobilized systemic antimicrobial immunity by inducing M1 macrophage and N1 neutrophil polarization, promoting DC maturation, and facilitating Th1 polarization of helper T cells, thereby suppressing bacterial infections. Thus, this ionic microbubble-mediated copper ion surge disrupts biofilm tolerance, enhances the bactericidal efficacy of copper ions, and elevates host antimicrobial immune responses, offering significant therapeutic benefits against biofilm-associated infections. By elucidating the complex interplay among copper ions, bacterial pathogens, and immune cells in overcoming biofilm tolerance homeostasis, this work provides a foundational framework for developing innovative copper-based therapies targeting recalcitrant biofilm infections.

**Figure 1:**
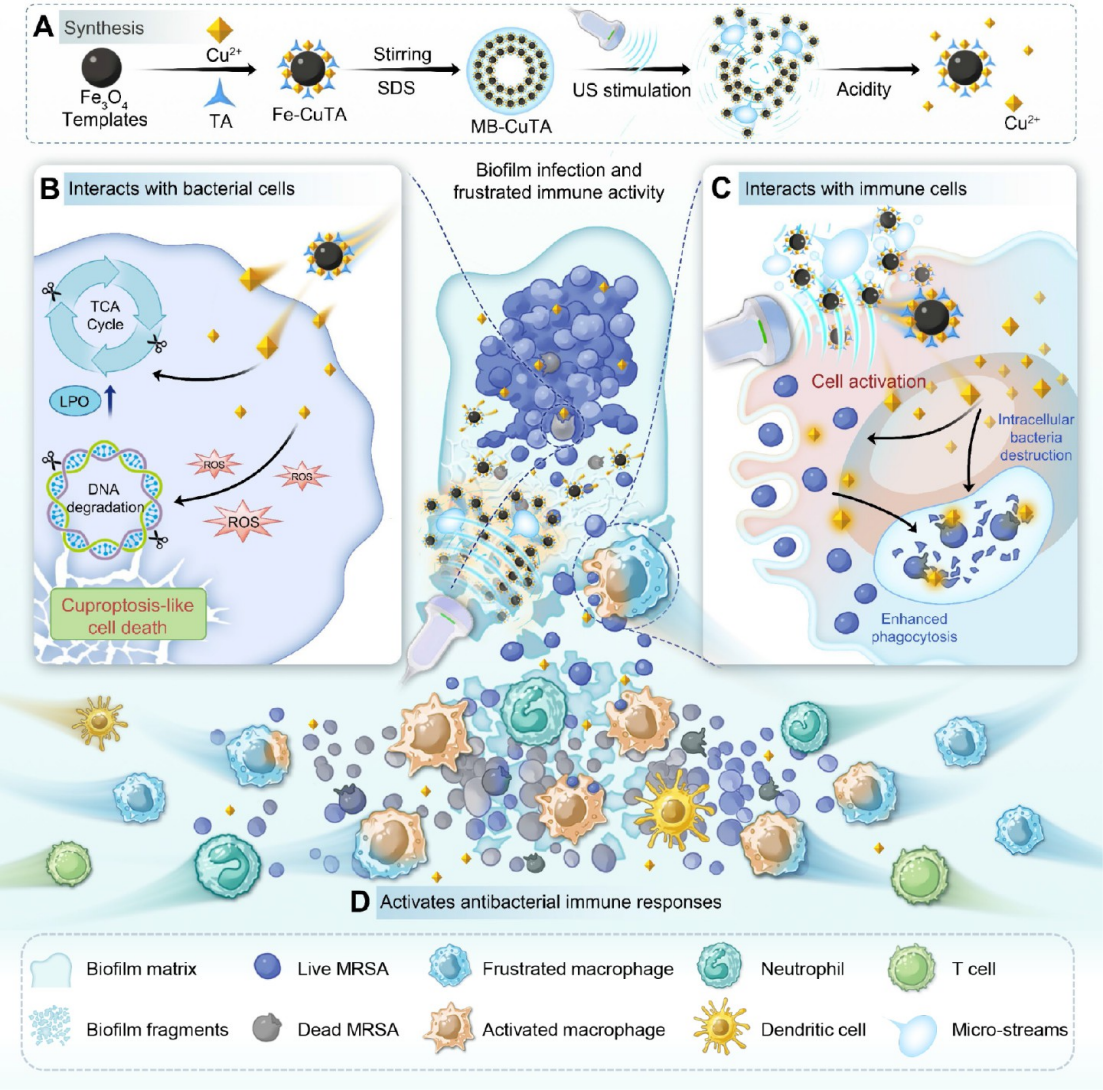
Design of US-stimulated MB-CuTA to medicate “copper ion surge” for the disruption of biofilm tolerance homeostasis. (A) Fabrication of MB-CuTA and its US-controlled release behavior. (B) Internalized copper ions mediate ROS production and disrupt the tricarboxylic acid (TCA) cycle, resulting in lipid peroxide (LPO) accumulation, thus triggering bacterial cuproptosis-like cell death. (C) With enhanced cellular uptake of Fe-CuTA nanoparticles, macrophages are activated and induced for intracellular bactericidal responses. (D) Activation of immune responses for defense against bacterial infection.

## 2 Results and Discussion

### 2.1 Synthesis and Characterization of Fe-CuTA Nanoparticles and MB-CuTA

Metal ion-cross-linked tannic acid (MITA), characterized by its environmentally friendly synthesis pathway, excellent biocompatibility, and remarkable adhesion properties, has been widely used for the development of diverse MITA-based adhesive materials.^26^ In this study, Fe_3_O_4_ nanoparticles were utilized as templates for the surface coating of copper ion cross-linked tannic acid (Fe-CuTA), and then Fe-CuTA nanoparticles were used for the self-assembly of microbubbles (MB) (Figure 2A). The synthesized Fe-CuTA exhibits a spherical morphology, with an average particle size of approximately 154.8 nm, which is marginally larger than that of the Fe_3_O_4_ nanoparticles (Figures 2B and S1). The coating of CuTA on Fe_3_O_4_ nanoparticles surface was further verified by elemental mapping images (Figures 2C and S2). Fe-CuTA was subsequently utilized for the preparation of MB-CuTA through mixing and agitating aqueous mixtures with sodium dodecyl sulfate (SDS) according to our previous work.^27,28^ Under agitation, the mixture can generate a gas core encapsulated with anionic surfactants, and the Fe-CuTA nanoparticles further assembled at the gas–liquid interface. As the gas diffuses, microbubbles gradually shrink until they are tightly packed. Scanning electron microscope (SEM) (Figure 2D) and bright-field microscopy images (Figure 2E) reveal the self-assembled morphology of the MB-CuTA, with an average diameter of approximately 17.36 μm (Figure 2F). Notably, the diameter of MB-CuTA is slightly smaller than that of MB, which could be attributed to the adhesive properties of CuTA (Figure S3). In addition, the elemental mapping images of MB-CuTA clearly demonstrated the homogeneous distribution of Fe, O, and Cu within MB-CuTA, confirming the successful synthesis of microbubble structures using Fe-CuTA nanoparticles (Figure S4). The concentrations of Fe and Cu in MB-CuTA were quantified using ultraviolet–visible (UV–vis) absorption spectrometry and inductively coupled plasma optical emission spectrometry (ICP-OES), respectively. By determining the number of MB-CuTA in various solution volumes, the amounts of Fe and Cu were calculated to be approximately 2.88 × 10^–9^ and 2.70 × 10^–10^ g per MB-CuTA (Figures 2G and S5).

**Figure 2:**
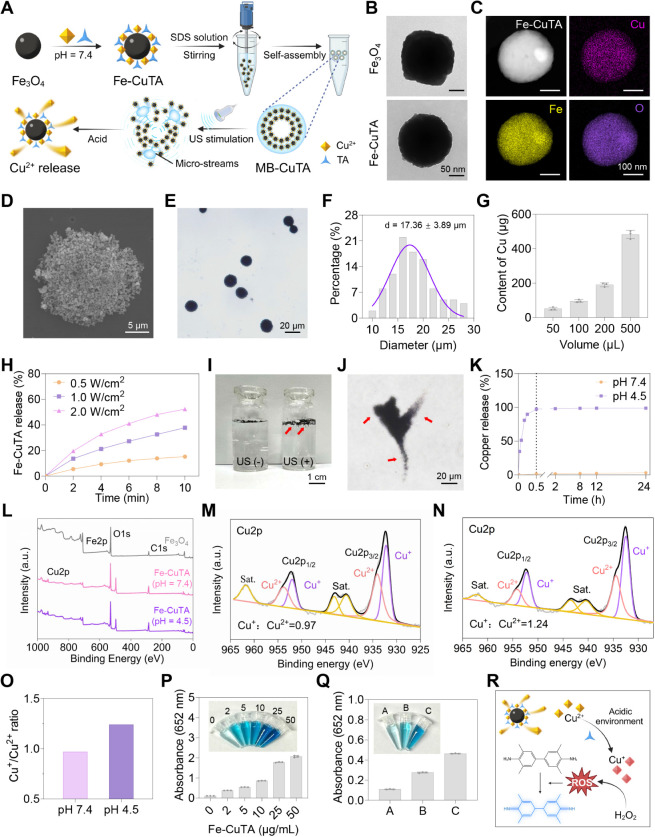
Preparation and characterization of Fe-CuTA and MB-CuTA. (A) Schematic illustration for the preparation of Fe-CuTA nanoparticles and MB-CuTA and drug release behavior. (B) TEM images of Fe_3_O_4_ nanoparticles and Fe-CuTA nanoparticles. Scale bar, 50 nm. (C) Elemental mapping of Fe-CuTA nanoparticles. Scale bar, 100 nm. (D) SEM images of MB-CuTA. Scale bar, 5 μm. (E) Representative bright-field microscopy image of MB-CuTA. Scale bar, 20 μm. (F) The diameter distribution of MB-CuTA (*n* = 100). (G) Content of Cu in different volumes of MB-CuTA dispersions (*n* = 3, mean ± s.d.). (H) Cumulative release profiles of Fe-CuTA nanoparticles from MB-CuTA under US stimulation with different powers (1 MHz, 50% amplitude) (*n* = 3). (I) Photographs and (J) microscopy images of MB-CuTA encapsulated in ultrasonic couplants with or without US stimulation (1 W/cm^2^, 1 MHz, 50% amplitude). Red arrows indicate the traces of microstreams generated by MB-CuTA under US stimulation. (K) Copper ion release profiles from Fe-CuTA nanoparticles under different pH values with increasing time (*n* = 3). (L) XPS spectrum of Fe_3_O_4_ nanoparticles and Fe-CuTA nanoparticles incubated in pH 7.4 or pH 4.5 acetic acid buffer for 15 min. (M,N) XPS spectra of Cu 2p in Fe-CuTA nanoparticles incubated in pH = 7.4 (M) or pH = 4.5 (N) acetic acid buffer for 15 min. (O) Ratios of Cu^+^/Cu^2+^ ions after incubation of Fe-CuTA nanoparticles under different pH. (P) Concentration-dependent ROS production of Fe-CuTA nanoparticles by using TMB as a substrate (CuTA basis) (*n* = 3, mean ± s.d.). Inset: Photographs of TMB solutions with different concentrations of Fe-CuTA nanoparticles. (Q) Peroxidase-like catalytic activity of MB-CuTA before and after US treatment (1.0 W/cm^2^, 1 MHz, 50% amplitude) by using TMB as a substrate (*n* = 3, mean ± s.d.). Inset: Photographs of TMB solutions with different experimental conditions (A: TMB + H_2_O_2_; B: MB-CuTA + TMB + H_2_O_2_; C: US-treated MB-CuTA + TMB + H_2_O_2_). (R) Schematic illustration of the acid-induced release and valence transition of copper ions and the peroxidase-like catalytic activity.

According to previous work, US stimulation can induce inertial cavitation of MB, leading to their gradual rupture and subsequent drug release.^29^ Inertial cavitation can induce the formation of microstreams, which have the potential to cause physical damage to the compact structure of bacterial biofilms or temporarily create pores in cell membranes, which may consequently enhance cell membrane permeability and facilitate drug uptake.^28,30^ As illustrated in Figure 2H, MB-CuTA can gradually release Fe-CuTA nanoparticles under US stimulation, and the extent of this controlled release was found to increase with higher US intensities. Furthermore, MB-CuTA can generate microstreams and facilitate deeper penetration of Fe-CuTA nanoparticles under US stimulation (Figure 2I,J). The released Fe-CuTA nanoparticles exhibit rapid copper ion release under acidic conditions with nearly complete liberation of the loaded copper ions occurring within 30 min in a pH 4.5 acetic acid buffer solution (Figure 2K). Since the acidic nature of the microbial infection microenvironment (pH 4.5–6.0),^31–34^ the permeating Fe-CuTA can induce a rapid copper ion release and penetration called “copper ion surge” within the bacterial biofilm matrix. More importantly, Cu^2+^ can be locally reduced to the Cu^+^ state in an acidic environment. X-ray photoelectron spectroscopy (XPS) analysis confirmed the elemental composition and oxidation states of copper within Fe-CuTA (Figures 2L–N and S6). The acidic environment significantly increases the ratio of Cu^+^/Cu^2+^ in Fe-CuTA nanoparticles, which may be attributed to the self-oxidation of TA to corresponding semiquinones (Figure 2O).^35^ The valence shift of the oxidation state of Cu ions in acidic environments makes it a promising peroxidase-like enzyme that can produce toxic ROS for antimicrobial applications. By using the 3,3′,5,5′-tetramethylbenzidine (TMB) as an indicator, the peroxidase-like activity of Fe-CuTA was evaluated by adding it to a solution containing hydrogen peroxide (H_2_O_2_) and TMB in an acidic acetate buffer. As the concentration of Fe-CuTA increased, it was observed that the absorption of TMB at 652 nm was obviously increased, which can be assigned to the generation of ROS during this process (Figures 2P and S7). Furthermore, in the absence of US stimulation, the peroxidase-like activity of MB-CuTA is not fully exerted (Figure 2Q), suggesting that controlled US exposure is essential for activating its catalytic efficacy. These findings confirmed that MB-CuTA is capable of generating mechanical stress and releasing Fe-CuTA under low-intensity US stimulation. Furthermore, Fe-CuTA can rapidly release substantial amounts of copper ions under acidic conditions, which undergo a valence state transition and induce ROS production (Figure 2R). Consequently, US-responsive MB-CuTA becomes a promising candidate as a copper ion delivery agent.

To further explore the potential biomedical applications, comprehensive evaluations of the *in vitro* cytotoxicity and hemolytic activity of MB-CuTA were conducted. The CCK-8 assay demonstrated that, despite the CuTA concentration in MB-CuTA reaching 62.5 μg/mL, its cytotoxicity toward RAW264.7 cells remained in a minimal state (Figure S8A). The cytotoxicity to RAW264.7 cells was further assessed *in vitro* using Calcein AM/propidium iodide (PI) staining. As illustrated in Figure S8B, there are almost no obvious PI fluorescent staining cells, indicating its low cytotoxicity. The erythrocyte hemolysis ratio was ∼8.7% when the concentration of CuTA reached 250 μg/mL, suggesting the low hemolysis of MB-CuTA (Figure S9).

### 2.2 Disruption of MRSA Biofilms by US-Stimulated MB-CuTA *In Vitro*

As illustrated in Figure S10A,B, Fe-CuTA nanoparticles showed an excellent antibacterial effect against planktonic bacteria. Even at a low dose, for instance, when calculated by the CuTA concentration of 12.5 μg/mL, it is capable of killing 99.998% of the MRSA bacteria, confirming the superior antibacterial efficacy of Cu ions against planktonic MRSA. However, due to the tolerance of bacterial biofilms, the antibacterial effect of the Fe-CuTA nanoparticles on biofilms is not satisfactory. Even a high level of Fe-CuTA nanoparticles (200 μg/mL) can only inactivate 1.12 log unit (92.42%) of bacteria in the MRSA biofilm (Figure S10C,D), which may distribute to the protection of biofilm extracellular matrix for limiting Fe-CuTA nanoparticle penetration.^36^ Therefore, it is crucial to disrupt the structure of the biofilm for enhancing the penetration of antimicrobial species.

Under US stimulation, MB-CuTA can generate microstreams by inertial cavitation, which may disrupt biofilms and subsequently enhance the penetration of Fe-CuTA into biofilms. As shown in Figure 3A,D, the biofilm in the MB-CuTA + US group exhibited the highest reduction of bacteria, with ∼4.7 log units (99.998%), while that in the Fe-CuTA + US group was only ∼1.4 log units (95.8%), indicating that US-stimulated biofilm disruption by MB-CuTA can efficiently break bacterial biofilm resistance and kill most of the bacteria. The 3D CLSM was further used for the observation of the living state of biofilms. The MB-CuTA with the US stimulation can minimize the thickness of the bacterial biofilm and the number of alive bacteria (Figure 3B,E). Fe-CuTA nanoparticles merely have a sterilization effect on the surface layer of the MRSA biofilm. US stimulation can loosen the structure of the biofilm to a certain extent, allowing Fe-CuTA to achieve a better antibacterial effect compared to that without US stimulation. The crystal violet staining of biofilms further verified this point (Figures 3C and S11A). The relative biofilm mass was quantified by solubilizing the crystal violet-stained biofilms, revealing that under US stimulation, MB-CuTA reduced over 60% of the biofilm matrix, while Fe-CuTA nanoparticles can only degrade less than 10% of the EPS (Figure 3F). The SEM images revealed that the MRSA biofilm was almost eliminated after treatment with MB-CuTA + US, leaving only a few shrunken and ruptured bacteria on the surface of the titanium plate (Figures 3G and S11B). Subsequently, the abundant release and permeation of copper ions within the acidic microenvironment of bacterial biofilms were detected by coppersensor-1, a probe that is selective and sensitive of copper­(I) ions (Figure S12). The addition of Fe-CuTA nanoparticles allowed for the detection of released copper ions, but their penetration was confined to the superficial biofilm layers. In contrast, MB-CuTA under US stimulation showed enhanced copper ion penetration throughout the entire biofilm matrix, leading to significant bacterial exposure to copper ions and ultimately causing a notable reduction in Hoechst-stained viable bacterial populations. With the addition of H_2_O_2_ (100 μM), the catalytic effect of Fe-CuTA nanoparticles within the biofilm microenvironment was further investigated by detecting ROS generation. 3D CLSM images revealed that the treatment of MB-CuTA + US also facilitated the generation of ROS throughout the entire layer of the biofilm, reaching down to its bottom (Figures 3H and S13). The generation of ROS contributed to the chemical degradation of the EPS of biofilms and further reduced the consumption or degradation of antibacterial species such as copper ions. These findings suggest that MB-CuTA with US stimulation can effectively disrupt the MRSA biofilm structure, enhance the permeability of Fe-CuTA nanoparticles, facilitate the diffusion of copper ions within the biofilm, and subsequently generate ROS for bacterial inactivation.

**Figure 3:**
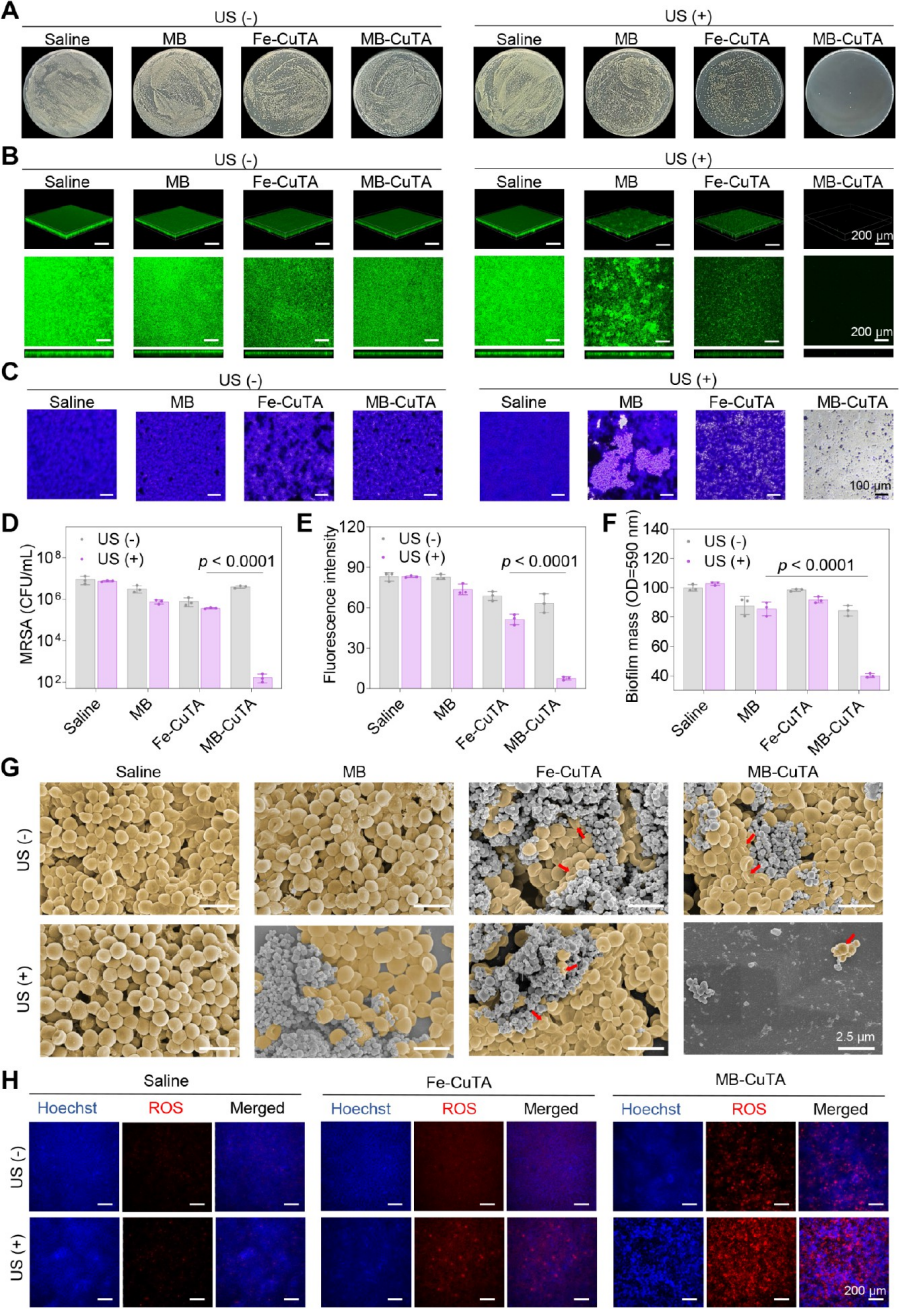
MRSA biofilm therapeutic efficacy after various treatments *in vitro*. (A) Photographs of MRSA colonies within MRSA biofilms in different groups (Fe_3_O_4_: 250 μg/mL; CuTA: 25 μg/mL; US: 1 MHz, 1.0 W/cm^2^, 50% amplitude, 10 min). (B) 3D confocal laser scanning microscope (CLSM) images of biofilms stained by Calcein-AM in different groups. Scale bar, 200 μm. (C) Microscope images of biofilms stained by crystal violet in different groups. Scale bar, 100 μm. (D) The number of live bacteria within MRSA biofilms calculated from (A) (*n* = 3, mean ± s.d.). (E) Fluorescence intensity of MRSA biofilms calculated from (B) (*n* = 3, mean ± s.d.). (F) Relative biofilm biomass measured by crystal violet-stained biofilms (*n* = 3, mean ± s.d.). (G) SEM images of MRSA biofilms in different groups. The pseudocolored yellow indicates the location of MRSA. Red arrows depict some of the shrunken bacteria. Scale bar, 2.5 μm. (H) CLSM images of biofilms in different groups stained by Hoechst and the ROS probe. Scale bar, 200 μm. Statistical differences were analyzed by a Student’s two-sided *t*-test (D–F).

### 2.3 Antibacterial Mechanism of Fe-CuTA Nanoparticles

To identify the mechanism by which Fe-CuTA nanoparticles kill bacteria, we further analyzed the RNA-sequencing results of MRSA before and after Fe-CuTA treatment. Correlation analysis confirmed the comparability of the three groups: Saline, Fe_3_O_4_ nanoparticle-treated MRSA (Fe_3_O_4_), and Fe-CuTA nanoparticle-treated MRSA (Fe-CuTA) (Figure S14A). Volcano plots revealed that the Fe-CuTA group exhibited 508 significantly differentially expressed genes (DEGs) in contrast to the Saline group, among which 209 genes were downregulated and 299 genes were upregulated (Figure 4A). Comparing with the Fe_3_O_4_ group, there were 762 DEGs in the Fe-CuTA group, with 340 genes being downregulated and 422 genes being upregulated (Figure 4B). Furthermore, only 93 DEGs were presented between Fe_3_O_4_ and Saline (Figure S14B).

**Figure 4:**
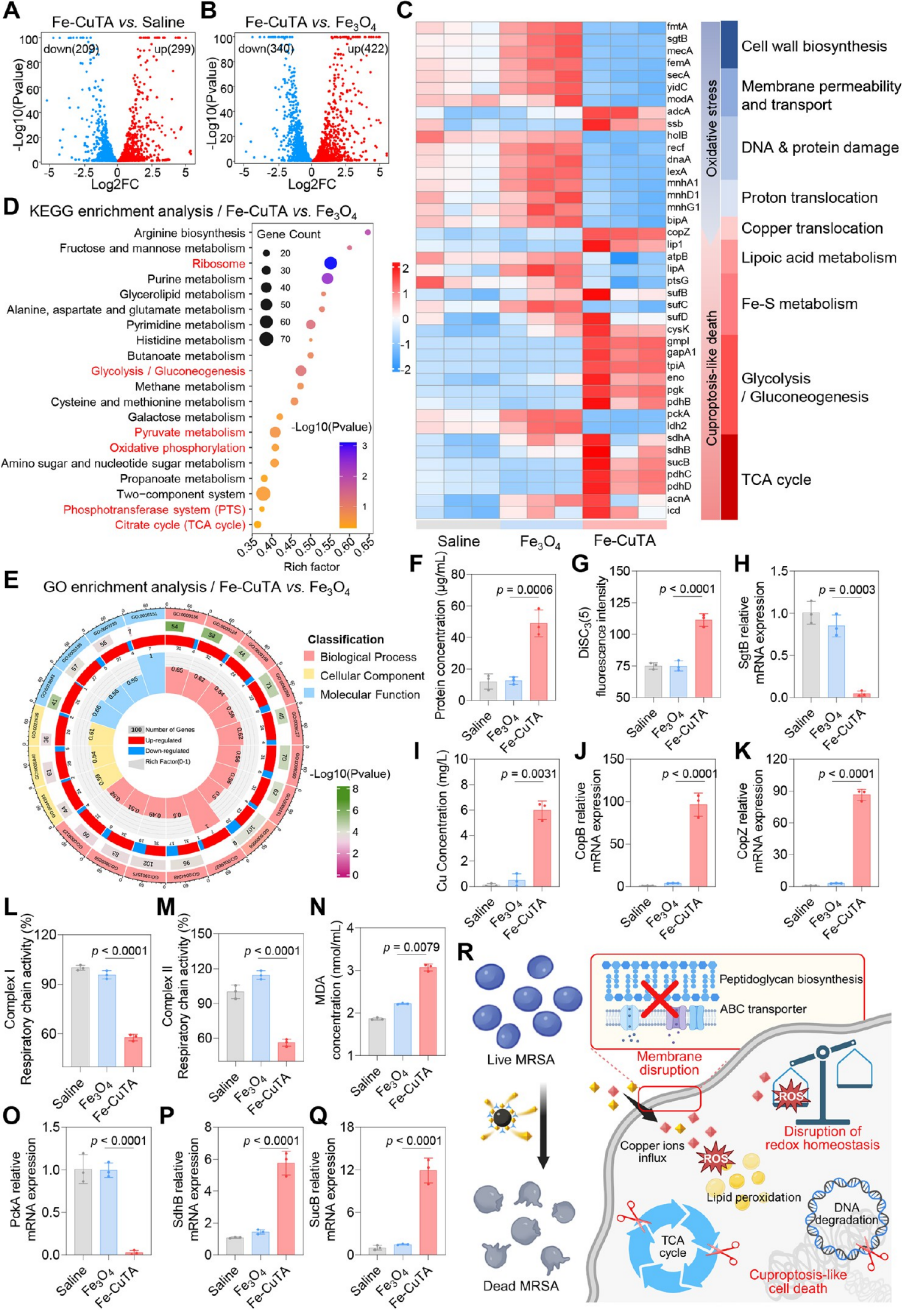
Differential gene expression of Fe-CuTA-treated MRSA and antibacterial mechanism. (A,B) Volcano plots of DEGs in MRSA treated with Fe-CuTA compared to Saline (A) and Fe_3_O_4_ nanoparticles (B) (blue: downregulated genes; red: upregulated genes). (C) Left: heatmap showing the differential expression of genes of interest. Right: the death pathways by ROS-induced oxidative stress and copper-induced cuproptosis-like death. (D) KEGG enrichment analysis scatter plot. (E) GO enrichment analysis. (F) BCA detection of bacterial protein leakage (*n* = 3, mean ± s.d.). (G) DiSC_3_(5) fluorescence intensity for bacterial membrane potential changes (*n* = 3, mean ± s.d.). (H) qPCR results of typical genes involved in cell wall composition (*n* = 3, mean ± s.d.). (I) The intracellular copper concentration was quantified using ICP-OES (*n* = 3, mean ± s.d.). (J,K) qPCR results of typical genes involved in copper ion transportation (*n* = 3, mean ± s.d.). (L,M) Activity of respiratory chain complex I (L) and II (M) of MRSA colonies (*n* = 3, mean ± s.d.). (N) MDA content of MRSA colonies (*n* = 3, mean ± s.d.). (O–Q) qPCR results of typical genes involved in the cuproptosis-like pathway (*n* = 3, mean ± s.d.). (R) Antibacterial mechanism diagram. Statistical differences were analyzed via a Student’s two-sided *t*-test (F–Q).

The above antibacterial results indicate that the main cause of bacterial death is CuTA on the surface of the Fe-CuTA nanoparticles. In order to further explore the mechanism, we analyzed these DEGs. According to the Kyoto Encyclopedia of Genes and Genomes (KEGG) and heatmap analyses, the significantly differentially expressed genes were primarily enriched in the tricarboxylic acid (TCA) cycle, phosphotransferase system, oxidative phosphorylation, pyruvate metabolism, glycolysis/glycolysis, ribosome, and metabolism of various amino acids and energy substances (Figures 4C,D and S14C). DEGs were also analyzed using Gene Ontology (GO) to determine their contribution to biological processes, cellular components, and molecular functions. Based on the enrichment analysis, metabolic processes, biosynthetic processes, catabolic processes, and ribosome activity were closely related to the mechanism through which Fe-CuTA nanoparticles kill MRSA (Figures 4E and S14D).

Based on the aforementioned comparison of the RNA sequencing outcomes among the three groups, the potential antibacterial mechanism of Fe-CuTA and validation experiments were presented as follows: (1) Oxidative stress led to severe disruption of the bacterial cell membrane, resulting in an increase in cell membrane permeability and protein leakage. The degree of protein leakage from MRSA after different treatments was detected using a BCA protein assay kit. After Fe-CuTA treatment, the leakage of bacterial proteins was significantly enhanced (Figure 4F). Then, we measured the membrane potential changes using the fluorescent dye DiSC_3_(5), a cyan dye whose fluorescence intensifies when the membrane potential dissipates.^37^ The bacterial membrane potential is one of the key factors for maintaining bacterial life activities, and as shown in Figure 4G, Fe-CuTA significantly altered the membrane potential compared with that of the Saline and Fe_3_O_4_ groups. Additionally, genes associated with peptidoglycan biosynthesis and ATP-binding cassette transporter (ABC transporter) were significantly downregulated. For instance, the level of expression of SgtB, which is one of the important genes related to peptidoglycan biosynthesis, significantly decreased (Figure 4H). (2) Bacterial cell membrane disruption subsequently led to a massive influx of copper ions. The copper content within the bacteria was determined by means of ICP-OES, confirming the significant increase of the copper concentration in the Fe-CuTA group (Figure 4I). Meanwhile, the genes related to copper ion-specific efflux and transport were significantly upregulated in MRSA, indicating that the amount of copper ions in MRSA cells was far beyond the physiological range (Figure 4J,K). The toxicity of excessive copper within bacterial cells and the generation of ROS caused severe damage to bacterial DNA and protein, resulting in a significant downregulation of genes related to DNA replication, mismatch repair, homologous recombination, and the two-component system. (3) More importantly, the genes related to pyruvate metabolism, the TCA cycle, and glycolysis/gluconeogenesis were substantially modulated, which indicated that the respiration and energy metabolism of bacteria were markedly obstructed. Coupled with the consideration of the enrichment of copper ions inside the bacteria, it implies that Fe-CuTA may lead to cuproptosis-like death of MRSA. Cuproptosis is a type of cell death, characterized by the restricted TCA cycling and peroxide accumulation.^38^ When excessive copper ions enter bacterial cells and transform into the highly toxic Cu^+^ state, cuproptosis-like death occurs, resulting in the aggregation of enzymes related to lipoic acid during the TCA cycle and causing disturbances in the function of Fe–S cluster proteins. Therefore, we validated the activities of respiratory chain complex I and complex II in different groups. As shown in Figure 4L,M, the activities of both respiratory chain complexes decreased in the Fe-CuTA group. The accumulation of malondialdehyde (MDA), a metabolic product related to lipid peroxides (LPO), was also determined. As depicted in Figure 4N, the results of the MDA assay indicated a significant increase in the level of LPO in the Fe-CuTA group. Ultimately, the restrained metabolites, through feedback regulation, modulated the expression of genes that regulate the TCA cycle (Figures 4O–Q and S14E–H). These findings suggest that Fe-CuTA nanoparticles are capable of eliciting intracellular toxic effects in bacteria analogous to eukaryotic cuproptosis, thereby inducing cuproptosis-like cell death through a similar underlying mechanism (Figure 4R).

### 2.4 Macrophage Activation by MB-CuTA *In Vitro* and Enhanced Intracellular Bactericidal Activity

Macrophages, as professional phagocytes, serve as the primary defense line of the body against infectious diseases. Floating bacteria released during biofilm infections and bacterial debris generated after US-responsive MB-CuTA treatment are phagocytosed by macrophages. Upon engulfing invading bacteria, macrophages employ a series of bactericidal mechanisms to effectively eliminate intracellular pathogens. However, certain bacteria can persist within macrophages and potentially trigger infection recurrence or systemic dissemination, particularly in the biofilm-infected microenvironment where macrophage function becomes frustrated.^12,39^ Therefore, strategies to activate macrophages for inducing their antimicrobial defenses are imperative.^40^ Metallic ions have been reported to stimulate macrophages to initiate or amplify broad-spectrum antimicrobial responses.^12,39,41^ For instance, macrophages can internalize copper ions to catalyze the production of hydroxyl radicals within the phagosomes. The released Fe-CuTA nanoparticles are supposed to induce macrophage immunomodulation via copper ion liberation, essentially activating macrophages to elicit a marked pro-inflammatory response.

To investigate the immunomodulatory effects of Fe-CuTA treatment on macrophages, total RNA from RAW264.7 after intervention with Saline, Fe_3_O_4_, or Fe-CuTA nanoparticles was collected and extracted for RNA-seq analysis. Overall, Fe-CuTA nanoparticles promote the polarization of macrophages toward the M1 pro-inflammatory phenotype (Figure 5A). Macrophages were found to be regulated by biofilms toward M2 polarization in the immune microenvironment of bacterial biofilm infections, thereby facilitating chronic infections. Macrophages of the M1 pro-inflammatory phenotype are crucial for combating bacterial infections and restricting immune escape through stronger phagocytic ability and increased secretion of pro-inflammatory cytokines. Volcano plots showed that the Fe-CuTA group exhibited 234 DEGs in contrast to the Saline treatment, among which 40 genes were downregulated and 194 genes were upregulated (Figure 5B). Compared with the Fe_3_O_4_ group, there were 186 DEGs in Fe-CuTA, with most of the genes upregulated (Figure 5C). Furthermore, only 30 DEGs were presented between Fe_3_O_4_ and Saline groups (Figure S15A), indicating that the variations in transcriptome genes are predominantly attributed to the regulation of copper ions. Heatmaps of differential gene expression indicated that upregulated genes, such as Cd80 and Tnf, were associated with the M1 phenotype of macrophages, while genes associated with macrophage M2 polarization, such as Il10 and Tgf, were significantly downregulated (Figure 5D). Figure 5E is a chord diagram comparing the Fe-CuTA and Fe_3_O_4_ groups, which indicates the relevant genes and KEGG pathways with close and multiple connections. Among these genes, several function as upstream signaling molecules that drive the polarization of macrophages toward the pro-inflammatory phenotype, with Jun, Relb, and Fos being prime examples. Meanwhile, there are another subset of genes that serve as pro-inflammatory factors and exhibit high expression levels in M1-polarized macrophages, notably Cxcl2, Ccl3, Cxcl10, Tnf, and Nfkb, among others. The relevant signaling pathways were also significantly enriched in the KEGG analysis of both the comparison between the Fe-CuTA group with the Fe_3_O_4_ group and with the Saline group (Figure S15B,C). Representative ones are the NF-kappa B signaling pathway, TNF signaling pathway, and MAPK signaling pathway, all of which mediate pro-inflammatory responses and enhance the phagocytic function of macrophages as well as the release of inflammatory mediators. Additionally, the enriched GO terms covered various aspects, mainly associated with the regulation of the immune system and response to stimulus (Figure S15D,E).

**Figure 5:**
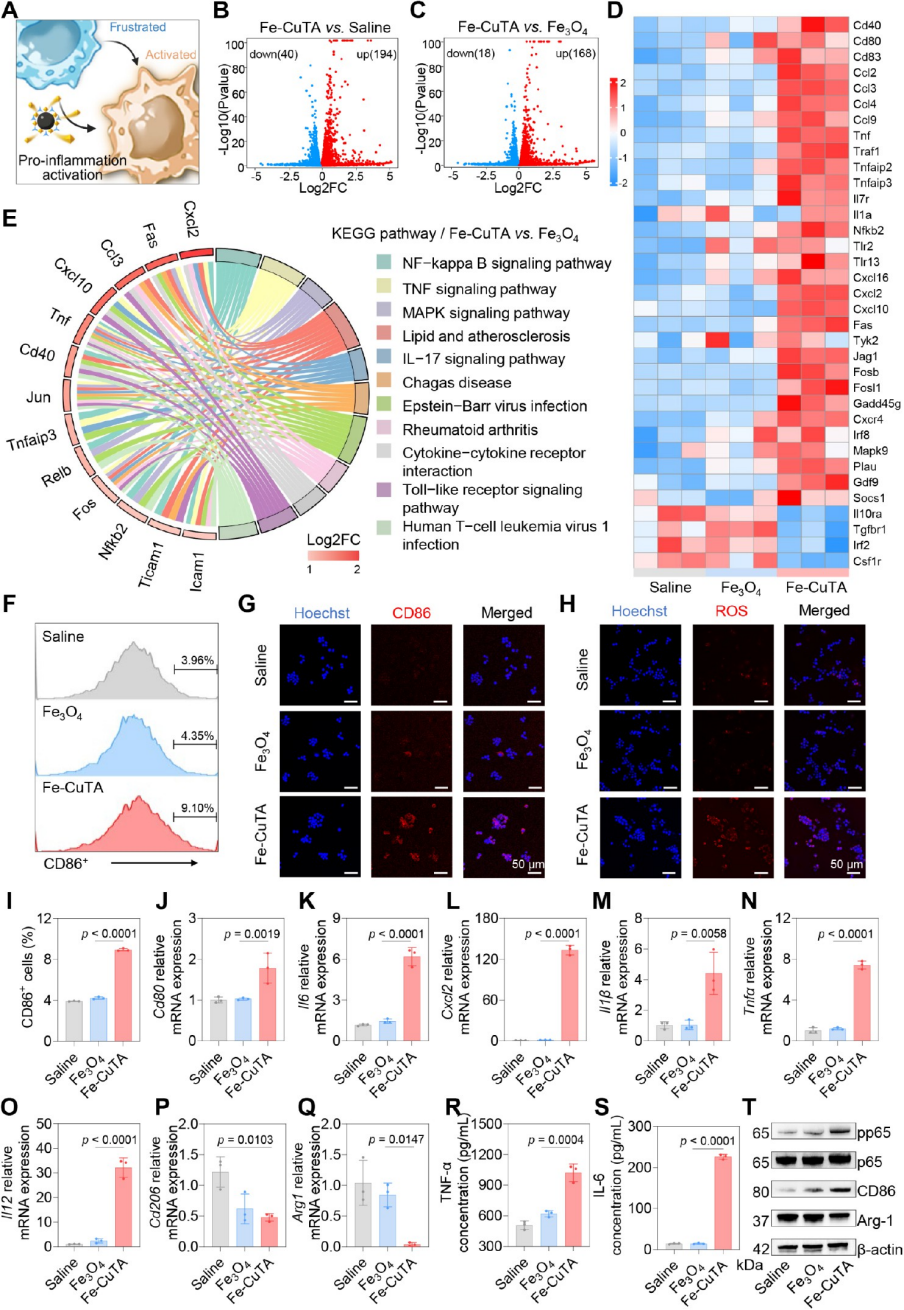
*In vitro* immune activation characterized by macrophage M1 polarization. (A) Schematic illustration of Fe-CuTA regulating macrophage polarization. (B,C) Volcano plots of DEGs in macrophages treated with Fe-CuTA compared to Saline (B) and Fe_3_O_4_ nanoparticles (C) (blue: downregulated genes; red: upregulated genes). (D) Heatmap showing the differential expression of genes of interest. (E) KEGG chord plot of differential gene enrichment pathways. (F) Representative FCM histograms for CD86^+^ cells in macrophages stimulated by Fe-CuTA. (G,H) Macrophage CD86 (G) and ROS (H) fluorescence staining images. Scale bar, 50 μm. (I) Proportions of CD86^+^ cells calculated from (F) (*n* = 3, mean ± s.d.). (J–Q) qPCR results of typical genes involved in macrophage polarization (*n* = 3, mean ± s.d.). (R,S) Secretion of TNF-α (R) and IL-6 (S) by macrophages in different groups (*n* = 3, mean ± s.d.). (T) Macrophage pp65, p65, CD86, and Arg-1 protein expression bands. Statistical differences were analyzed by a Student’s two-sided *t*-test (I–S).

To validate macrophage activation into the M1 pro-inflammatory phenotype, the surface marker CD86, representative of M1 macrophage polarization, was first subjected to staining detection. The flow cytometry (FCM) and CLSM showed that the Fe-CuTA treatment group exhibited a significantly high expression of CD86, whereas there was a negligible difference between Fe_3_O_4_ and the control groups (Figure 5F,G,I). Meanwhile, the level of ROS within macrophages increased significantly, indicating that copper ions similarly induced a redox imbalance in macrophages (Figure 5H). As dedicated phagocytes, the elevation of the oxidative stress level within macrophages not only facilitates the clearance of intracellular bacteria but also promotes their M1 polarization.^42^ Figure 5J–Q presents the qPCR results for assessing the mRNA expression levels of inflammatory biomarkers. In contrast to the Fe_3_O_4_ group and the Saline group, upon treatment with Fe-CuTA nanoparticles, the expression levels of pro-inflammatory cytokines, such as *Cd80*, *Il6*, *Cxcl2*, *Il1β*, *Tnfα*, and *Il12*, were significantly elevated, indicating the polarization of macrophages into the M1 phenotype. Meanwhile, the level of mRNA expression related to M2 polarization (such as *Cd206* and *Arg1*) decreased significantly. In addition, we measured the levels of cytokines secreted by macrophages using ELISA, functionally verifying the pro-inflammatory effect of Fe-CuTA nanoparticles (Figure 5R,S). Western blotting (WB) experiments further confirmed the pro-inflammatory phenotype of macrophages at the protein level, revealing increased pp65 and CD86 protein expression and decreased Arg1 protein expression (Figures 5T and S16). All of the aforementioned results indicated the copper ion-mediated immune activation effect represented by macrophage M1 polarization through Fe-CuTA treatment. This metal ion-triggered pro-inflammatory response in macrophages could be further validated through subsequent intracellular bacterial eradication experiments.

Subsequently, RAW264.7 macrophages infected with MRSA were treated with Fe_3_O_4_ or Fe-CuTA nanoparticles and then analyzed by colony counting to examine the intracellular antibacterial effect. As shown in Figure 6A–C, the Fe-CuTA nanoparticles internalized by macrophages and the subsequently released copper ions nonetheless manifested excellent efficacy in combating intracellular bacteria, with an inhibition rate reaching up to 99.995%. This antibacterial effect was diminished upon the use of a specific copper chelator, bathocuproinedisulfonic acid (BCS), demonstrating the critical role of copper ions in killing both intracellular and extracellular bacteria (Figure S17). The results of *in situ* transmission electron microscope (TEM) imaging can more intuitively reveal that the macrophages in the Fe-CuTA treatment group have phagocytosed a greater number of bacteria (Figure 6D). Moreover, the majority of the intracellular bacteria exhibit a vacuolated morphology accompanied by cytoplasmic leakage, suggestive of their death due to copper ion intoxication (Figure S18). The enhanced phagocytic function of the activated macrophages was also demonstrated by CLSM imaging. RAW264.7 macrophages were cocultured with Saline, Fe_3_O_4_, or Fe-CuTA nanoparticles for 12 h. Then, the agents were washed away, and green fluorescent MRSA was added. The CLSM images showed that macrophages preactivated by Fe-CuTA nanoparticles exhibited enhanced phagocytosis (Figure 6E). Activated macrophages can phagocytose planktonic bacteria, which also assisted to further enhance the antibacterial efficiency for the MRSA biofilm after treated by MB-CuTA with US stimulation. The therapeutic effects of Fe-CuTA nanoparticles for MRSA with the addition of macrophages were further evaluated. As depicted in Figure 6F,G, the bacterial number in the Fe-CuTA + macrophage group was decreased by approximately 5.68 log units (99.9998%), which was higher than that in the Fe-CuTA group (4.40 log units, 99.996%). Meanwhile, the number of bacteria in the Fe_3_O_4_ + macrophage group exhibited a limited change compared with that in the Fe_3_O_4_ group. These results indicated that macrophages can be activated by Fe-CuTA nanoparticles for pro-inflammation effects and can assist the bactericidal effect on MRSA.

**Figure 6:**
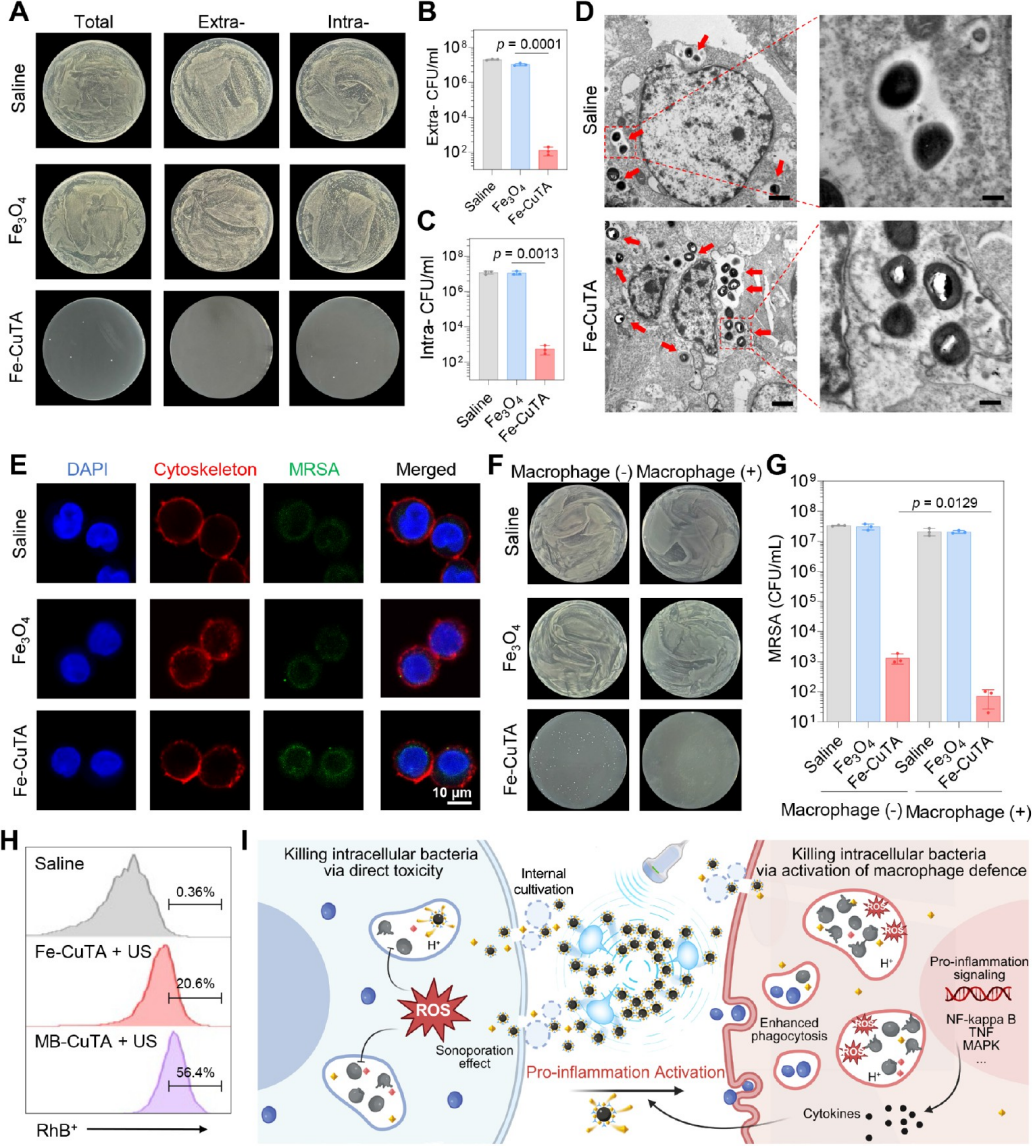
Enhanced intracellular antibacterial activity. (A) Photos of total, extracellular, and intracellular MRSA colonies with different treatments. (B,C) The number of live extracellular (B) and intracellular (C) MRSA calculated from (A) (*n* = 3, mean ± s.d.). (D) *In situ* TEM images of the MRSA-infected macrophages after incubation with Saline and Fe-CuTA for 4 h. Red arrows indicate intracellular MRSA. Scale bar, 1 μm (left) and 300 nm (right). (E) Macrophage phagocytosis staining images. Red fluorescence represents the cytoskeleton. Green fluorescence represents MRSA. Scale bar, 10 μm. (F,G) Photographs (F) and number (G) of live bacteria with or without the addition of macrophages (10^6^/well) (*n* = 3, mean ± s.d.). (H) Macrophage uptake of Fe-CuTA nanoparticles stained with Rhodamine B for 4 h (US: 1 MHz, 0.5 W/cm², 50% amplitude, 5 min). (I) Scheme for enhanced macrophage function for intracellular bacteria elimination. Statistical differences were analyzed by a Student’s two-sided *t*-test (B,C,G).

Moreover, compared to traditional antimicrobial agents, which have difficulty in effectively penetrating the immune cells, microbubbles can temporarily create pores in cell membranes under US stimulation by inertial cavitation. The process of mechanically opening the cell membrane using microbubbles and US is known as sonoporation, which helps to deliver more therapeutic agents into cells.^25^ MB-CuTA with US stimulation can enhance the macrophage phagocytosis of Fe-CuTA nanoparticles, resulting in enhanced intracellular accumulation of copper ions. As depicted in Figure 6H, FCM was employed to detect the uptake of Fe-CuTA nanoparticles dyed with Rhodamine B (RhB) in the form of microbubbles or not by macrophages under US stimulation. This indicates that the cavitation of microbubbles under US stimulation can promote the uptake of released Fe-CuTA nanoparticles by macrophages. The internalized copper ions can be directly toxic to intracellular bacteria and, at the same time, break immune suppression and activate macrophage-mediated antimicrobial inflammation (Figure 6I). This dual function substantially enhances the ability of macrophages to engulf and eradicate intracellular bacteria, thereby accelerating the clearance of infections and reducing the risk of recurrence.

### 2.5 Treatment of Biofilm-Associated Implant Infection *In Vivo*

To determine the therapeutic effect of US-responsive MB-CuTA on bacterial biofilms *in vivo*, a mouse model of implant-associated infections (IAIs) caused by MRSA biofilms was established. IAIs are associated with significant morbidity and mortality in clinical practice. Substantial research has been devoted to preventing and treating IAIs, and the prognosis is intricately linked to the effective eradication of biofilms.^43–45^ In the mouse model of subcutaneous IAIs, titanium sheets were first implanted into the thighs of mice for 1 day. Then, the mice were infected with MRSA for 2 days, thereby constructing the implant infection model induced by MRSA biofilm. Different agents were subcutaneously injected into infected tissue, followed by the US stimulation in US-treated groups, and the healing process of infected tissues was observed (Figure 7A). First, we verified the penetration effect of US-stimulated MB-CuTA within subcutaneous tissue. As depicted in Figure 7B, US stimulation facilitated better penetration and accumulation of Fe-CuTA nanoparticles pumped by microbubbles within biofilm-infected tissues. This phenomenon can be attributed to two key aspects: the inherent excellent tissue penetrability of ultrasound itself, which gives ultrasonic antibacterial therapy a distinct advantage over other therapies such as photothermal therapy (PTT), coupled with the cavitation effect induced by microbubbles under US stimulation, which not only facilitates the disruption of bacterial biofilm barriers and enhances cellular uptake but also increases drug penetration within tissues.^46,47^ Post-3 days of treatment, it was observed that the MB-CuTA + US group manifested a substantially limited area of infection. Conversely, in the remaining groups, infected areas persisted and bacterial abscesses had developed, encircling the titanium sheets (Figure S19). Bacterial plate counting was then carried out within the biofilm-infected tissues surrounding the titanium sheets. As shown in Figure 7C,D, the number of live MRSA was reduced by ∼3.04 log units (99.91%) in the infected tissue for the MB-CuTA + US group compared with that in the Saline group, which was much higher than that of the Fe-CuTA + US group (96.2%). SEM images of the titanium implant further demonstrated that the MRSA biofilm almost vanished in the MB-CuTA + US group (Figure S20).

**Figure 7:**
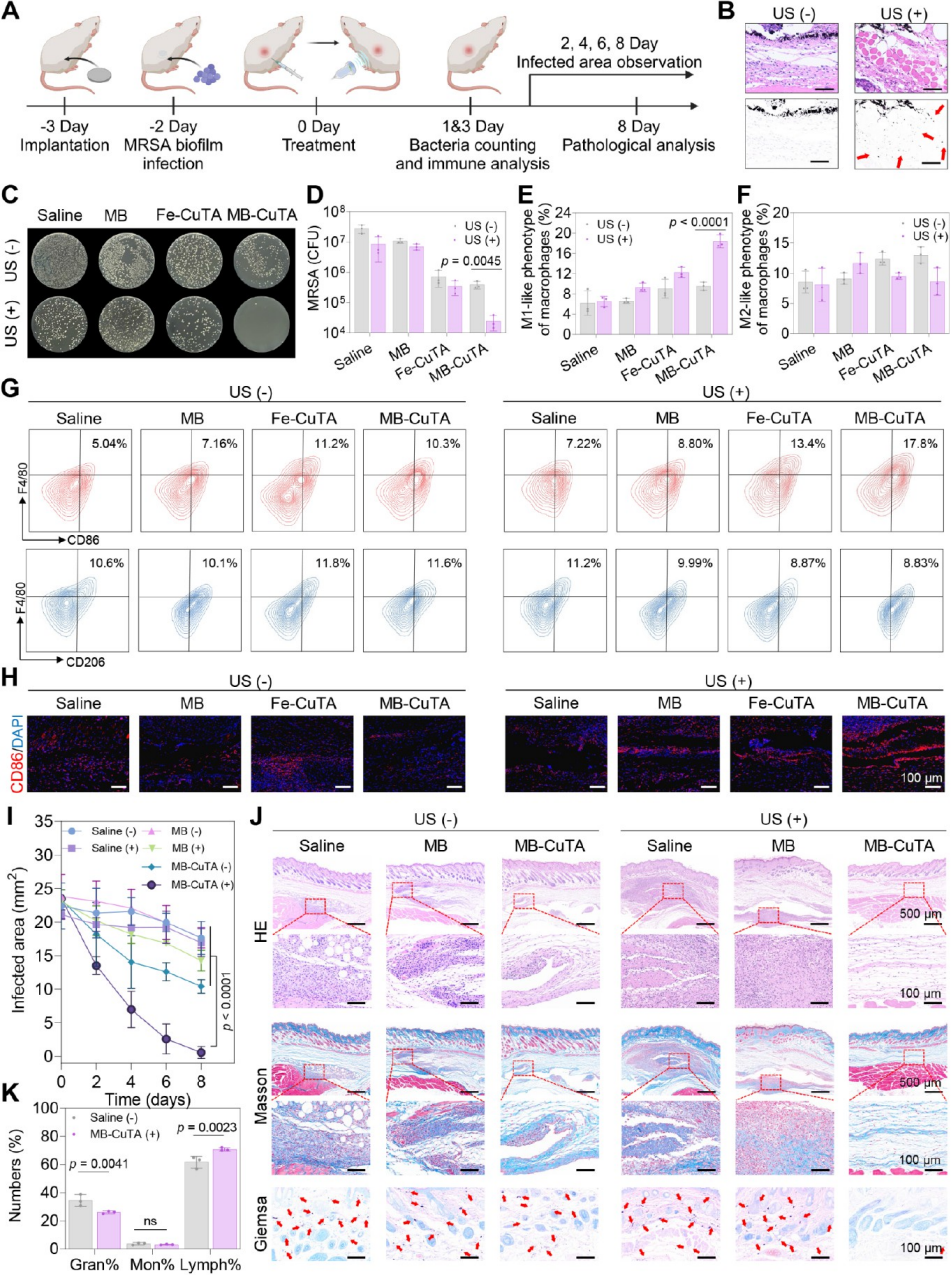
Treatment of MRSA biofilm-associated implant infection *in vivo*. (A) Schematic illustration of the strategies for the treatment of biofilm-induced implant infection in a mouse model. (B) Microscope images of the slice of the infected tissue stained by HE after various treatments. Red arrows indicate penetrated Fe-CuTA nanoparticles. Scale bar, 50 μm. (C,D) Pictures of MRSA colonies in plate (C) and the corresponding colony number of live MRSA (D) in infected tissues in different groups for 3 d (*n* = 3, mean ± s.d.). (E–G) Polarization of M1-like macrophages (CD86^+^) (E) and M2-like macrophages (CD206^+^) (F) in different groups calculated from FCM analysis (G) for 3 d (*n* = 3, mean ± s.d.). (H) Immunofluorescent staining for CD86 across various treatment groups for 3 d. Scale bar, 100 μm. (I) Average infection area growth curves of MRSA biofilm-infected mice in different groups during 8 days (*n* = 3, mean ± s.d.). (J) HE, Masson, and Giemsa staining images of infected tissues after various treatments for 8 d. Scale bars, 500 and 100 μm. (K) Gran%, Mon%, and Lymph% detected by blood routine examination after various treatments for 8 d (*n* = 3, mean ± s.d.). Statistical differences were analyzed by a Student’s two-sided *t*-test. ns, no significant difference (D–F,I,K).

The immunomodulatory effect of MB-CuTA on IAIs caused by MRSA biofilms *in vivo* was further assessed. As depicted in Figure S21, the results of FCM revealed that the infiltration of macrophages (CD11b^+^F4/80^+^) was approximately 4.42% in the MB-CuTA + US group, which was higher than those in the Fe-CuTA + US treatment group (∼2.95%) and the Saline + US group (∼1.21%). It indicates that under US stimulation, MB-CuTA enables Fe-CuTA nanoparticles and the released copper ions to penetrate more deeply, leading to the recruitment of a greater number of macrophages at the infected site. More importantly, the polarization of the M1 type among these macrophages was significantly enhanced (F4/80^+^CD86^+^). Compared with the Saline group, approximately 3-fold more macrophages presented the M1 phenotype in the MB-CuTA + US group (Figure 7E–G). In addition, no significant trend of alterations in the polarization of M2-like macrophages was observed. Moreover, the immunofluorescence images further confirmed that, compared with the Saline group and the Fe-CuTA group, a greater number of CD86^+^ macrophages in the MB-CuTA + US group accumulated within the infected tissues surrounding the implant, while no obvious change was observed in CD206^+^ macrophages (Figures 7H and S22). Similarly, the antibacterial and pro-inflammatory activation effects came to the fore preliminarily as early as the first day post-treatment (Figure S23). All these results suggest that the “copper ion surge” mediated by US-responsive MB-CuTA disrupts the bacterial biofilm tolerance homeostasis through copper ion-mediated bacterial cuproptosis-like death and cellular immune activation, thereby contributing to the efficient elimination of IAIs.

Eventually, on the eighth day after treatment, the MB-CuTA + US group had the smallest infected area, almost achieving complete elimination of the biofilm infection (Figures 7I and S24). The images of HE and Masson-stained slices of the infected tissues revealed that varying degrees of inflammatory cell infiltration occurred in each group, except for the MB-CuTA + US group. Regardless of whether there is US stimulation, both the Saline group and the MB group had obvious subcutaneous abscesses containing a large number of inflammatory cells and damaged tissue cells. In contrast, no obvious inflammation was found around the implant site in the MB-CuTA + US group, and good tissue repair was observed. Moreover, Giemsa staining confirmed that a considerable number of bacteria persisted in all groups except the MB-CuTA + US group, further substantiating the induced antibacterial efficiency of MB-CuTA under US stimulation *in vivo* (Figure 7J). In addition, an analysis was conducted on the peripheral blood of mice post-8 days of treatment. For the MB-CuTA + US group, the inflammation abated, with the percentage of granulocytes reducing and the lymphocytes involved in adaptive immunity increasing (Figure 7K).

Furthermore, the *in vivo* biocompatibility of MB-CuTA under US stimulation was evaluated. No significant differences were observed in serum biochemical parameters or hematological indices between the mice in the US-stimulated MB-CuTA group and the control group (Figure S25A–I). Histopathological examination via HE staining revealed no notable damage or inflammation in major organs, suggesting that MB-CuTA exhibits low toxicity *in vivo* (Figure S25J). Additionally, previous studies have raised concerns that physical or chemical dispersal of bacterial biofilms might potentially induce acute bacterial dissemination.^48^ Therefore, after treating biofilms with microbubbles and US, the bacterial plate counts were performed on mouse blood 1 day later. The results showed no observed bacterial entry into the bloodstream, thereby supporting the favorable safety profile of this therapeutic modality (Figure S26A,B). In addition, *in vitro* results demonstrated that while effective microbubble-mediated disruption of bacterial biofilms carries a risk of bacterial dispersion into the surrounding environment, subsequent exposure to copper ions in the MB-CuTA + US group successfully eradicated all released planktonic bacteria, thereby significantly mitigating the potential risk of secondary transmission (Figure S26C,D).

### 2.6 *In Vivo* Antibacterial Effect and Immune Activation of MB-CuTA in Peritonitis Infection Model

To verify whether MB-CuTA could reprogram the host immune response for enhanced treatment of bacterial infections, the peritonitis model induced by MRSA was established by intraperitoneal injection of MRSA into mice. Subsequently, the antibacterial effect was evaluated by intraperitoneal administration of the different agents with or without US stimulation (Figure 8A). After 16 h of treatment, the total, extracellular, and intracellular MRSA CFUs in the peritoneal fluid were determined (Figure 8B). Due to the recruitment of a substantial number of phagocytes by bacterial infection, the proportion of bacteria remaining extracellular in the peritoneal fluid is relatively low. Moreover, treatment with MB-CuTA + US eliminated approximately 98.8% of the residual extracellular bacteria (Figure S27). It is worth noting that for intracellular bacteria, the released Fe-CuTA nanoparticles with enhanced uptake by peritoneal macrophages under US stimulation enable copper ions to exhibit excellent antibacterial properties within infected cells. However, MB-CuTA without US treatment failed to effectively inhibit intracellular bacteria (Figure 8C). The released copper ions, along with the subsequent disruption of redox homeostasis, can also stimulate the activation of peritoneal macrophages in the similar manner as in the subcutaneous tissue. As illustrated in Figure 8D,E, the MB-CuTA + US group elicited approximately 3-fold higher M1-type polarization of peritoneal macrophages compared to the Saline group, thereby enhancing macrophage phagocytosis and elimination of intracellular bacteria. Similarly, there was no significant change in the relative number of CD206^+^ macrophages (Figure S28A,B). The activation of macrophages results in the production of a substantial array of cytokines, which facilitate the recruitment of neutrophils to the peritoneal cavity.^49^ Neutrophils serve as one of the primary lines of the innate immune defense by identifying bacteria via pattern recognition receptors (PRRs).^50^ Meanwhile, neutrophils can be induced to polarization states analogous to those of macrophages: N1-polarized neutrophils, similar to M1 macrophages, display pro-inflammatory responses with enhanced phagocytic and antimicrobial capabilities.^51^ As is illustrated in Figures 8D,F and S28A,C, FCM analysis of peritoneal cells indicated a significant rise in the relative quantity of neutrophils expressing the N1 marker CD54 in the MB-CuTA + US group, while neutrophils expressing the N2 marker CD170 remained relatively low. The copper ion-induced immunophenotypic changes of these specialized phagocytes suggest their elevated intracellular oxidative stress levels and high efficiency of intracellular bacteria clearance.

**Figure 8:**
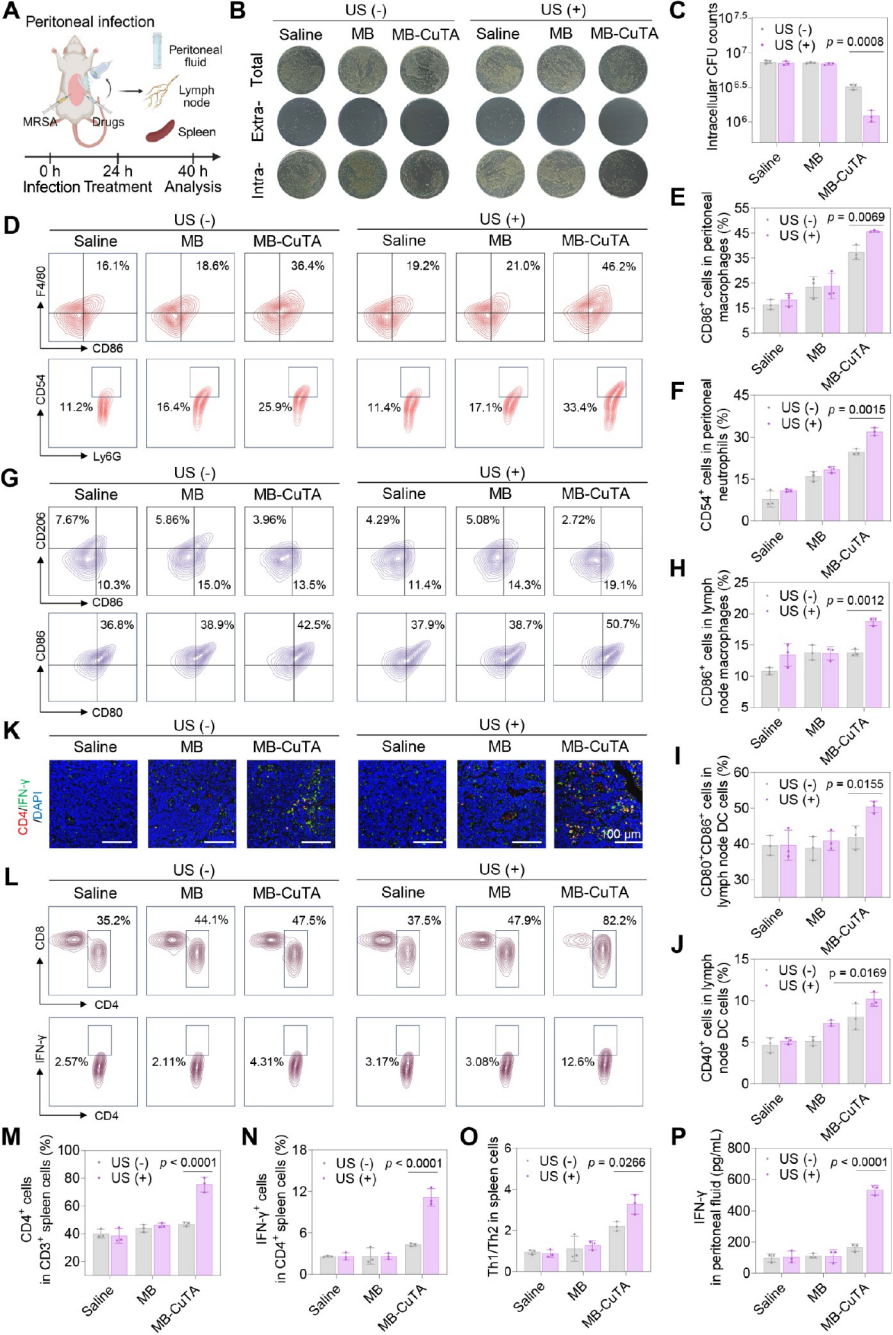
Intracellular antibacterial efficiency and immune activation of MB-CuTA in the mouse peritonitis model. (A) Schematic illustration of the MRSA-infected peritonitis model. (B,C) Photographs (B) and intracellular CFUs (C) in peritoneal lavage fluid determined 16 h after the different treatments (*n* = 3, mean ± s.d.). (D) FCM analysis of M1-like macrophages (CD11b^+^F4/80^+^CD86^+^) and N1-like neutrophils (CD45^+^Ly6G^+^CD54^+^) after different treatments in peritoneal lavage fluid. (E,F) Quantification of M1-like macrophages (E) and N1-like neutrophils (F) calculated from (D) (*n* = 3, mean ± s.d.). (G) FCM analysis of M1-like (CD11b^+^F4/80^+^CD86^+^) and M2-like (CD11b^+^F4/80^+^CD206^+^) macrophages and activated DCs (CD11c^+^CD80^+^CD86^+^) after different treatments in mesenteric lymph nodes. (H,I) Quantification of M1-like macrophages (H) and activated DCs (I) calculated from (G) (*n* = 3, mean ± s.d.). (J) Quantification of activated DCs (CD11c^+^CD40) by FCM analysis (*n* = 3, mean ± s.d.). (K) Immunofluorescence images of mesenteric lymph nodes from peritonitis mice stained with CD4 and IFN-γ antibodies after various treatments. Scale bar, 100 μm. (L) FCM analysis of Th cells (CD3^+^CD8^–^CD4^+^) and Th1 cells (CD3^+^CD8^–^CD4^+^IFN-γ^+^) after different treatments in spleen tissues. (M,N) Quantification of Th cells (M) and Th1 cells (N) calculated from (L) (*n* = 3, mean ± s.d.). (O) The ratio of Th1/Th2 cells after different treatments in spleen cells (*n* = 3, mean ± s.d.). (P) Levels of IFN-γ in peritoneal lavage fluid (*n* = 3, mean ± s.d.). Statistical differences were analyzed by a Student’s two-sided *t*-test (C,E,F,H–J,M–P).

As critical peripheral immune organs, the lymph nodes and spleen play an indispensable role in the progression of peritoneal infection. Furthermore, studies have indicated that an increased copper metabolism is necessary during inflammatory infections to guarantee the functioning of the immune system, and there is increasing evidence of a connection between copper supplementation and the disruption of immune tolerance.^52–54^ After the MB-CuTA treatment (with sufficient copper supplementation), FCM analysis was carried out to assess immune cells in the mesenteric lymph nodes and spleen, which represented the systemic immune status of the mice. As shown in Figures 8G,H and S29A, macrophages in mouse mesenteric lymph nodes were also activated and showed more M1-type polarization. In addition, the proportion of mature DCs in mesenteric lymph nodes was higher, with the upregulation of costimulatory molecules on the surface of DC cells, such as CD80, CD86, and CD40 (Figures 8G,I,J and S29B). Mature DCs can not only stimulate the inflammatory response to combat bacterial infection but also effectively uptake bacterial antigens and present exogenous bacterial antigens to helper T lymphocytes (Th cells), shaping the subsequent adaptive immune response.^55^ Th cells possess a variety of antimicrobial immune functions, such as the generation of effector cytokines and the coordination of other immune cells and nonimmune cells, which is beneficial for the balance of the immune response and the elimination of bacteria within the body. As illustrated in Figure 8K, the immunofluorescence-stained slices of lymph nodes revealed a significantly higher number of CD4^+^ cells in the MB-CuTA + US group, suggesting an enhanced mobilization of Th cells. Moreover, a large amount of IFN-γ is secreted by CD4^+^ cells in the MB-CuTA + US group, which might be associated with the Th1 phenotype differentiation of Th cells. IFNγ^+^ Th1 cells are associated with cellular immunity against intracellular pathogens. However, bacterial infection, especially in biofilm form, can develop immune evasion strategies that disrupt Th1 lymphocyte differentiation and activation by impairing antigen-presenting cell (APC) functionality.^56,57^ This immunomodulatory effect leads to diminished production of IFN-γ and other critical cytokines, ultimately compromising the bactericidal capacity of effector cells, such as macrophages. The spleen serves as a critical immune organ, where T cells colonize and initiate immune responses. As shown in Figures 8L,M and S30, the proportion of Th cells increased substantially not only in splenic T lymphocytes, but also in all collected cells of the spleen after copper supplementation by US-stimulated MB-CuTA treatment. More importantly, according to FCM results, copper ion stimulation led to a large increase in the proportion of IFN-γ-secreting Th1 cells, similar to Th cell differentiation in mesenteric lymph nodes (Figure 8L,N). Furthermore, the Th1/Th2 ratio was significantly elevated (Figure 8O). Notably, Th2 cells are characterized by IL-4 secretion, and a predominant immune environment of Th2 cells may weaken Th1-mediated cellular immunity, thereby potentially facilitating biofilm formation and immune evasion.^58,59^ Finally, the concentrations of IFN-γ in both the peritoneal fluid and serum were significantly increased by the copper ion stimulation (Figures 8P and S31). The IFN-γ produced by Th1 cells can not only enhance phagocyte activity but also further promote differentiation of Th1 cells via positive feedback mechanisms, thereby intensifying the inflammatory response to combat bacterial infection.^57^ In addition, these therapeutic effects and immune activation outcomes were significantly potentiated after treatment by MB-CuTA with US stimulation, further confirming the ability of the microbubble structure to enhance tissue penetration and cellular uptake of copper ions under US.

## 3 Conclusions

In this study, we developed Fe-CuTA nanoparticles that self-assemble into acoustically responsive microbubbles (MB-CuTA), offering a strategy to disrupt biofilm tolerance homeostasis. The microbubble constructs demonstrate distinctive acoustic responses: when exposed to low-intensity ultrasound, they exhibit stable oscillatory contractions, whereas surpassing a certain threshold induces inertial cavitation. This phenomenon generates localized microstreams accompanied by potent mechanical forces capable of overcoming the recalcitrant barrier of the bacterial biofilm matrix. This ultrasonic cavitation mechanism for biofilm barrier disruption demonstrates significantly enhanced efficiency and safety compared to alternative approaches (Table S1). Specifically, compared with laser-assisted therapy, this approach demonstrates superior tissue penetration, and in contrast to enzymatic degradation approaches, it exhibits enhanced stability. More importantly, this microbubble generates a substantial accumulation of copper ions after US stimulation, resulting in a surge of copper ions in infected tissues, which plays a critical role in disrupting biofilm tolerance. The biofilm matrix acts as both a physical barrier that restricts antimicrobial penetration and a biochemical shield that interferes with host immune recognition, leading to antimicrobial tolerance and immune evasion. For bacterial biofilms, the enhanced penetration of copper ions into deeper biofilm layers circumvents the extracellular polymeric matrix barrier, allowing for direct and extensive interaction with bacteria. This increased bioavailability of copper ions disrupts bacterial metabolic homeostasis, leading to the cuproptosis-like death of MRSA. For immune cells, the sonoporation-mediated cellular uptake of copper ions not only reactivates frustrated macrophages to enhance the clearance of intracellular bacteria but also counteracts biofilm-induced immune suppression. Bacteria actively modulate immune defenses by inhibiting macrophage inflammatory polarization, suppressing antigen presentation, and dysregulating neutrophil responses, leading to persistent infections. The surge of copper ions in infected tissues overcomes these immune defenses by inducing M1 macrophage polarization and Th1 cell differentiation, activating N1-polarized neutrophils, and promoting dendritic cell maturation, thereby reestablishing a robust immune response. This process reshapes the host–pathogen interface, ensuring bacterial eradication not only through direct bactericidal activity but also through immune reprogramming. This multimodal mechanism, driven by an ionic microbubble-mediated copper ion surge, effectively integrates direct antimicrobial action with host immune modulation, presenting a promising therapeutic approach to overcoming biofilm tolerance and ultimately achieving efficient clearance of biofilm-associated infections.

## 4 Experimental Section

### 4.1 Materials

Tannic acid (TA, ≥99.5%), copper­(II) chloride dihydrate (CuCl_2_·H_2_O, ≥99%), sodium dodecyl sulfate (SDS, ≥98.5%), and 3,3′,5,5′-tetramethylbenzidine (TMB, ≥99%) were obtained from Sigma-Aldrich. Ferric oxide nanoparticles (Fe_3_O_4_) were purchased from Alfa Aesar (USA). Calcein/PI and CCK-8 kits were purchased from Beyotime (China). CellROX Oxidative Stress Reagent was purchased from Thermo Fisher (USA). NF-κB p65, Phospho-NF-κB p65, Arginase-1, CD86, and β-actin antibodies were bought from Cell Signaling Technology (USA). The enzyme-linked immunosorbent assay (ELISA) kits for interleukin-6 (IL-6), tumor necrosis factor-α (TNF-α), and interferon-γ (IFN-γ) were acquired from MultiSciences. The FCM antibodies, which include anti-CD86, anti-CD206, anti-F4/80, anti-CD11b, anti-CD3, anti-CD4, anti-CD8, anti-CD11c, anti-CD80, anti-CD40, anti-CD11b, anti-Ly6G, anti-CD54, anti-CD170, anti-IFN-γ, and anti-IL4, were bought from BioLegend (CA, USA).

### 4.2 Preparation of Microbubbles Assembled by Fe-CuTA Nanoparticles

Prior to the fabrication of MB-CuTA, Fe-CuTA was fabricated by dispersing 10 mg of Fe_3_O_4_ nanoparticles in 20 mL of ultrapure water. Subsequently, 18 μL of tannic acid (3 mg/mL) solution and 175 μL of CuCl_2_·2H_2_O (10 mg/mL) solution were added under a vortex. The pH of the solution was gradually raised to approximately 7.4 by using 1 M NaOH solution under vortex. Finally, Fe-CuTA nanoparticles were obtained through centrifugation (10 000 rpm, 5 min) and rinsed with ultrapure water three times to remove unreacted tannic acid and Cu^2+^.

The MB-CuTA were prepared in accordance with previous studies. First, the aqueous dispersions of Fe-CuTA nanoparticles were fabricated with the aid of US. Subsequently, SDS solution (10 mM, 150 μL) and Fe-CuTA nanoparticles (10 mg/mL, 400 μL) were mixed and homogenized at 20 000 rpm for 3 min to form MB-CuTA. The mixture was stored overnight at room temperature for the close packing of Fe-CuTA nanoparticles. Finally, MB-CuTA were separated by magnet and washed three times with deionized water. The morphology of MB-CuTA was observed through an optical microscope and a scanning electron microscope. The content of Fe_3_O_4_ in MB-CuTA was calculated with the aid of a spectrophotometer. The concentration of Cu in MB-CuTA was measured by means of ICP-OES.

### 4.3 Component Release from Fe-CuTA and US-Stimulated MB-CuTA

The release of copper ions from Fe-CuTA nanoparticles was studied at a time-dependent gradient in two different pH conditions (pH = 7.4, 4.5). 2.5 mg of Fe-CuTA nanoparticles was dispersed in 4 mL of acetic acid buffers (0.2 M) with pH values of 7.4 and 4.5, respectively. Then, the solution was incubated in a shaker at 37 °C and vibrated for 180 rpm. At specified intervals, 500 μL of supernatant was collected through centrifugation (10 000 rpm, 5 min) to determine the release amount of copper ions by ICP-OES.

The release of Fe-CuTA nanoparticles from MB-CuTA was examined under US stimulation. MB-CuTA was dispersed in H_2_O and stimulated with US (1 MHz, 50% amplitude) at different frequencies (0.5, 1.0, 2.0 W/cm^2^) for varying times. After MB-CuTA was removed using the magnet, the released Fe-CuTA nanoparticles in the solution were quantified through spectrophotometry.

### 4.4 Detection of ROS Generation *In Vitro*

The peroxidase-like catalytic activity of Fe-CuTA nanoparticles and MB-CuTA, with or without US stimulation, was evaluated using TMB as a substrate. Different amounts of materials in 0.2 M NaAc-HAc buffer (pH 4.5; 500 μL) were mixed with TMB (100 mM, 10 μL), and H_2_O_2_ was further added to the reaction solution to achieve a final concentration of 1 mM. The A652 value of the reaction mixture was measured after 5 min at 37 °C in the dark.

### 4.5 Treatment of MRSA Biofilms *In Vitro*

The MRSA biofilm was incubated with Saline, MB, Fe-CuTA, and MB-CuTA (Fe_3_O_4_ NPs: 0.25 mg/mL; CuTA: 0.025 mg/mL), stimulated by US (1 MHz, 1.0 W/cm^2^, 50% amplitude, 10 min), and then incubated for 12 h. After different treatments, the MRSA biofilms were harvested, gently washed with saline three times to remove the substances, and then dispersed in saline for 5 min under an ultrasonic cleaner (10^8^ W, 10% amplitude) to fully disperse the biofilms. Finally, the standard plate counting method was employed to quantify the number of viable MRSA in the biofilms. The MRSA dispersion was serially diluted 10-fold. 100 μL of the diluted MRSA dispersion was plated into LB agar plates, and the plates were incubated inverted at 37 °C for 24 h. Finally, the number of colonies was multiplied by the dilution factor to calculate the number of viable MRSA in the original solution. The fluorescence imaging of the treated MRSA biofilms was observed through the adoption of Calcein-AM staining and CLSM. To quantify the biofilm biomass, the MRSA biofilms after different treatments were washed with saline and then fixed with formalin for 30 min. After being naturally dried, the fixed biofilms were stained with crystal violet for 20 min. Subsequently, they were washed with saline three times and finally decolorized with acetic acid for 30 min. The absorbance of all samples was measured at 570 nm, and the relative biofilm biomass of the treated biofilms was calculated. The MRSA biofilms on titanium sheets were fixed with 2.5% glutaraldehyde and then dehydrated by an ethanol gradient before being photographed by SEM.

The generation of ROS in MRSA biofilms was detected. MRSA biofilms were cultivated in confocal culture dishes and then treated with Saline, Fe-CuTA, and MB-CuTA (Fe_3_O_4_ NPs: 0.25 mg/mL; Cu: 0.025 mg/mL). After the addition of H_2_O_2_ (100 μM), the biofilms were stimulated by US (1 MHz, 1.0 W/cm^2^, 50% amplitude, 10 min) and subsequently incubated for 2 h. After being stained with CellROX (Deep Red, a ROS probe) and Hoechst for 30 min, the generation of ROS within the MRSA biofilms was observed by CLSM.

### 4.6 Transcriptome Analysis of MRSA

Saline, Fe_3_O_4_, and Fe-CuTA (Fe_3_O_4_ NPs: 0.25 mg/mL; CuTA: 0.025 mg/mL) were used to treat MRSA for 4 h. Bacteria were collected in the logarithmic growth phase and immediately frozen in liquid nitrogen with three parallel controls set for each group. The NEBNext Ultra II RNA Library Prep Kit for Illumina was used to build sequencing library, completed by Nanjing Personal Biotechnology Co. Ltd. (China). The products were purified and quantified by an Agilent high-sensitivity DNA assay on the Agilent Bioanalyzer 2100 system. The sequencing library underwent sequencing by an Illumina NovaSeq 6000 platform. FastQC was used for the raw data quality control. The DESeq2 was used to analyze expression quantification results, identifying DEGs with |log_2_FC |≥1 and *p*-value <0.05.

### 4.7 Transcriptome Analysis of RAW264.7 Macrophage

RAW264.7 cells were seeded at a density of 1 × 10^6^ and then cultured overnight. Subsequently, Saline, Fe_3_O_4_, or Fe-CuTA (Fe_3_O_4_ NPs: 0.25 mg/mL; CuTA: 0.025 mg/mL) was added, and the mixture was incubated for 8 h. After being washed three times with PBS, the samples were collected using TRIzol reagent for transcriptome sequencing analysis. The construction of the cDNA library and sequencing with the NovaSeq 6000 platform (Illumina) were completed by Nanjing Personal Biotechnology Co., Ltd. (China)

### 4.8 Assessment of Macrophage Polarization

RAW264.7 cells were incubated with Saline (control), Fe_3_O_4_, or Fe-CuTA (Fe_3_O_4_ NPs: 0.25 mg/mL; CuTA: 0.025 mg/mL) for 8 h. The phenotype markers of RAW264.7 were analyzed through staining with anti-CD86 for FCM and CLSM analysis. Additionally, the culture supernatant was collected to detect the secretion of TNF-α and IL-6 using ELISA kits. The mRNA levels indicating the macrophage phenotype were detected by RT-qPCR after extracting RNA from RAW264.7 with different treatments, which was confirmed by WB analysis for the evaluation of related protein expression levels. Furthermore, intracellular ROS generation was determined by using the CellROX Deep Red reagent as a probe.

### 4.9 Macrophage-Assisted Bactericidal Effect

To assess the antibacterial effects of different agents in combination with macrophages, the MRSA after various treatments was further incubated with RAW264.7 (10^6^ cells per well) in DMEM (containing 10% FBS) under a 5% CO_2_ atmosphere at 37 °C for 24 h. All samples were collected, and the live MRSA was calculated by using the standard plate counting method. Each group carried out three biological replicates as parallel experiments.

### 4.10 Assessment of Cellular Uptake Activity of Nanoparticles

RAW264.7 cells were seeded in 6-well plates at a density of 1 × 10^6^ and then incubated overnight. Fe-CuTA or MB-CuTA was stained with Rhodamine B (RhB) overnight. After adding Saline, Fe-CuTA, or MB-CuTA, US stimulation was performed, and then the cells were incubated for 4 h. After being washed with PBS three times, the RAW264.7 cells were fixed by 4% paraformaldehyde (PFA), and the cell uptake was detected using a Sony SA3800 flow cytometer.

### 4.11 Animals and Ethical Statement

Female Balb/c mice (∼20 g, 6–8 weeks old) were purchased from Shanghai Sippe-Bk Lab Animal Co., Ltd. All surgical procedures and postoperative care of the animals were performed according to the guidelines of the Institutional Animal Care and Use Committee. The animal experiment was approved by the Animal Ethical and Welfare Committee of Nanjing University (IACUC-D2303104).

### 4.12 Treatment of MRSA Biofilm-Induced Implant Infection *In Vivo*

Balb/c mice were anesthetized, shaved, and disinfected to establish a mouse implant infection model induced by MRSA biofilms. Sterile titanium plates (with a diameter of 6 mm) were implanted into the right thighs of the mice and allowed to grow for 1 d. Subsequently, 50 μL of MRSA at a concentration of 10^8^ CFU/mL dispersed in LB (containing 1% glucose) was subcutaneously injected into the implants. After 2 days of infection, biofilms were formed. The infected mice were randomly divided into 8 groups. 50 μL of diverse drugs (Saline; MB: 1 mg/mL Fe_3_O_4_ NPs; Fe-CuTA: 1 mg/mL Fe_3_O_4_ NPs and 0.1 mg/mL CuTA; MB-CuTA: 1 mg/mL Fe_3_O_4_ NPs and 0.1 mg/mL CuTA) was injected subcutaneously, respectively. Among them, 4 groups were subjected to US stimulation (1 MHz, 1.0 W/cm^2^, 50% amplitude, 10 min). Each group conducted three biological replicates as parallel experiments.

One day after the treatment, the subcutaneous tissues were collected for HE staining to observe the drug penetration situation. The infected tissues of the mice were collected 1 or 3 days after various treatments. The infected tissues were cut into small pieces. After being sonicated for 5 min, the live bacteria were analyzed by the standard plate counting method. Subsequently, at 37 °C, the small pieces of infected tissues were digested with type I collagenase (2 mg/mL) and deoxyribonuclease (100 μg/mL) for 30 min. Then, the digested tissues were filtered through a 70 μm filter, and the single-cell suspension was collected to be stained with FITC antimouse CD11b, PerCP-Cy5.5 antimouse F4/80, PE antimouse CD86, and APC antimouse CD206. The stained cells were then measured by using a SA3800 FCM and analyzed with FlowJo software. For immunofluorescence staining, the infected tissues were collected and fixed 3 d after various treatments. All tissues were stained with primary antibodies (anti-CD86 antibody and anti-CD206 antibody), fluorescently labeled secondary antibodies, and DAPI after being sectioned. All slices were scanned using an Olympus IX81.

During the entire treatment period, the infection site of each mouse was monitored every other day for calculation of the infection area. The body weight of each mouse was also recorded. After 8 days of treatment, the mice were sacrificed. All of the infected tissues were collected and fixed with formalin for pathological examination. After being embedded in paraffin and sliced, HE, Masson, and Giemsa staining were performed. Additionally, peripheral blood was collected to assess the immune response.

### 4.13 *In Vivo* Antibacterial Activity for Intracellular MRSA in Peritonitis Model

The *in vivo* anti-intracellular bacteria effect was detected by using a peritonitis model. Briefly, the MRSA bacterial solution (5 × 10 ^7^ CFU/mouse, 100 μL) was intraperitoneally injected into mice. After incubation for 24 h, the mice were randomly divided into 6 groups (*n* = 3). Different drugs were respectively injected intraperitoneally once for treatment (Saline; MB: 2.5 mg/kg Fe_3_O_4_ NPs; MB-CuTA: 2.5 mg/kg Fe_3_O_4_ NPs and 0.25 mg/kg CuTA) with or without US stimulation (1 MHz, 1.0 W/cm^2^, 50% amplitude, 10 min). 16 h after the treatment, the mice were sacrificed after being intraperitoneally injected with 2 mL of cold PBS. The peritoneal fluid was collected to measure the total CFUs, extracellular CFUs, and intracellular CFUs. One-third of the peritoneal fluid was used to quantify the total CFUs. The supernatant was collected by centrifuging another third of the peritoneal fluid to quantify the extracellular CFUs. One-third of the peritoneal fluid was treated with lysostaphin (15 μg/mL) to kill the extracellular MRSA. Then, the cells were lysed with PBS containing 0.1% BSA and 0.1% Triton X-100, and the intracellular CFUs were quantitatively detected.

### 4.14 Assessment of the Immune Response in the Peritonitis Model

After treatment, the peritoneal lavage fluid was collected. A single-cell suspension was obtained after being washed with PBS to conduct FCM antibody staining. For peritoneal macrophages, cells were stained with anti-CD11b-FITC, anti-F4/80-PercpCy5.5, anti-CD86-PE, and anti-CD206-APC. For peritoneal neutrophils, cells were stained with anti-CD45-FITC, anti-Ly6G-PE, anti-CD54-APC, and anti-CD170-PE-Cy7. Furthermore, the mesenteric lymph nodes and spleens of the infected mice were harvested and dissected into small pieces. After undergoing enzymatic digestion for 30 min, a single-cell suspension was obtained by passing it through a 70 μm filter. For lymph node cells, macrophages were stained with anti-CD11b-FITC, anti-F4/80-PercpCy5.5, anti-CD86-PE, and anti-CD206-APC. DC cells were stained with anti-CD11c-AF700, anti-CD80-PECy7, anti-CD86-APC, and anti-CD40-PE. For splenic cells, FCM antibody staining was performed after erythrocyte lysis, and T cells were stained by anti-CD3-APC, anti-CD4-FITC, anti-CD8-PercpCy5.5, anti-IFN-γ-APC, and anti-IL4-PE-Cy7.

### 4.15 Statistical Analysis

Data are presented as mean values ± s.d. The statistical difference between two groups was determined by the Student’s *t*-test. One-way or two-way analysis of variance was used for multiple comparisons. The analysis was performed on Prism 10 (GraphPad Software). Results are presented as *p* < 0.05, which was considered significant.

## Supplementary Material



## Notes

Figure S1. The diameter distribution of nanoparticles. Figure S2. Elemental mapping of Fe_3_O_4_ nanoparticle. Figure S3. The diameter of MB. Figure S4. Elemental mapping images of MB-CuTA nanoparticles. Figure S5. (A) Content of Fe in different volumes of MB-CuTA dispersions. (B) The number of MBs in different volumes of MB-CuTA dispersions. Figure S6. XPS spectra of Fe 2p. Figure S7. Concentration-dependent ROS production of (A) CuTA nanoparticles and (B) Fe_3_O_4_ nanoparticles by using TMB as a substrate. Figure S8. Evaluations of the *in vitro* cytotoxicity. Figure S9. Histopathological examination. Figure S10. The therapeutic efficiency of Fe-CuTA. Figure S11. (A) General view of biofilm stained by crystal violet in 96-well plate. (B) SEM images of MRSA biofilms in different groups. Figure S12. 3D CLSM images of biofilm stained by Hoechst (blue) and coppersensor-1 (red). Figure S13. 3D CLSM images of MRSA biofilm stained by Hoechst (blue) and ROS probe (red). Figure S14. (A) Correlation analysis between sample. (B) Volcano plots of DEGs in MRSA treated with Fe_3_O_4_ nanoparticles compared to Saline. (C) KEGG enrichment analysis scatter plot. (D) GO enrichment analysis. (E to H) qPCR results of typical genes involved in cuproptosis-like pathway. Figure S15. (A) Volcano plots of DEGs in macrophages treated with Fe_3_O_4_ nanoparticles compared to Saline. (B and C) KEGG enrichment analysis. (D and E) GO enrichment analysis. Figure S16. Quantitative analysis of (A) pp65, (B) p65, (C) CD86 and (D) Arg1 protein expression. Figure S17. Copper inhibitor reverses antibacterial effects against extracellular/intracellular MRSA of Fe-CuTA. Figure S18. (A) In situ elemental mapping images of the infected macrophages. Atomic percentage of (B) Cu, (C) Fe and (D) O. Figure S19. Photographs of bacterial biofilms around titanium disks in different groups for 3 d. Figure S20. SEM images of implants collected from infected tissues in different groups for 3 days. Figure S21. (A) FCM analysis of macrophages (CD11b^+^F4/80^+^) infiltration at the infection site and (B) the quantitative analysis. Figure S22. Immunofluorescent staining for CD206 across various treatment groups for 3 d. Figure S23. (A) Pictures of live MRSA colonies in infected tissues in different groups for 1 d. (B) FCM analysis of M1-like and M2-like macrophages post 1 d. (C) Corresponding colony number of live MRSA calculated from (A). (D) Quantitative analysis of M1-like phenotype and (E) M2-like phenotype macrophages. Figure S24. (A) Weight change curves of infected mice after various treatments. (B) Representative photos of implant infection after treatments for 8 d. Figure S25. *In vivo* toxicity evaluation of MB-CuTA. Figure S26. Assessment of bacterial dissemination risk *in vivo* and *in vitro*. Figure S27. (A) Total and (B) extracellular CFUs in peritoneal lavage fluid determined 16 h after the different treatments. Figure S28. (A) FCM analysis of M2-like macrophages and N2-like neutrophils after different treatments in peritoneal lavage fluid. (B) Quantification of M2-like macrophages and (C) N2-like neutrophils. Figure S29. (A) Quantification of M2-like macrophages in lymph nodes. (B) FCM analysis of CD40^+^ DCs in lymph nodes. Figure S30. Quantification of Th1 cells in all spleen lymphocytes after different treatment. Figure S31. Levels of IFN-γ in serum. Table S1 The performance of materials for biofilm disruption in the literature. Table S2. List of detailed primer sequences for MRSA mRNA. Table S3. List of detailed primer sequences for macrophage mRNA (PDF)

## Abbreviations

MB-CuTA: ionic microbubbles
EPS: extracellular polymeric substance
Fe-CuTA: copper-tannic acid-coated Fe_3_O_4_ nanoparticles
US: ultrasound
ROS: reactive oxygen species
MRSA: Methicillin-resistant Staphylococcus aureus
DC: dendritic cells
Th: helper T cells
MB: self-assembly of microbubbles
UV–vis: ultraviolet–visible
ICP-OES: inductively coupled plasma optical emission spectrometry
XPS: X-ray photoelectron spectroscopy
TMB: 3,3′,5,5′-tetramethylbenzidine
PI: propidium iodide
DEGs: differentially expressed genes
KEGG: Kyoto Encyclopedia of Genes and Genomes
TCA: tricarboxylic acid
GO: Ontology
MDA: malondialdehyde
LPO: lipid peroxides
FCM: flow cytometry
WB: Western blotting
IAIs: implant-associated infections
PRRs: pattern recognition receptors
