# Supplementary material for: Disrupting Biofilm Tolerance by Ionic Microbubble-Mediated Copper Ion Surge for Infection Clearance
Source: ACS Nano. 2025 Jul 31;19(31):28624–43. doi: 10.1021/acsnano.5c08035 (PMC12356198; doi:10.1021/acsnano.5c08035)
Supplement: Supplementary file 1 [file nn5c08035_si_001.pdf]

Supplementary Information

Disrupting Biofilm Tolerance by Ionic Microbubble-mediated Copper Ion Surge for Infection Clearance

Xiaoye Li,[1] Qiang Li,[1] Ao He,[1] Meng Dang,[1] Yu Zhang,[1] Minjin Wang,[1] Qinhong Sun,[2] Zhuo Dai,[1] Meng Ding,[1] Jingben Zheng,[1] Yongbin Mou,[1] Weijun Xiu,[3*] and Heng Dong[1*]*

[1]Nanjing Stomatological Hospital, Affiliated Hospital of Medical School, Institute of Stomatology, Nanjing University, 30 Zhongyang Road, Nanjing, 210008, China.

[2]Department of Thoracic Surgery, Jiangsu Cancer Hospital & Jiangsu Institute of Cancer Research & Nanjing Medical University Affiliated Cancer Hospital, Jiangsu Key Laboratory of Molecular and Translational Cancer Research, Nanjing, 210009, China

[3]Department of Biomedical Engineering (BME), National University of Singapore, 15 Kent Ridge Cres, Singapore 119276, Singapore

*Corresponding author. yongbinmou@nju.edu.cn (Y. M.ou), wjun_xiu@nus.edu.sg (W. Xiu), dongheng90@smail.nju.edu.cn (H. Dong)

CONTENTS

Fig. S1. The diameter distribution of nanoparticles.

Fig. S2. Elemental mapping of Fe_3O_4 nanoparticle. Scale bar is 100 nm.

Fig. S3. The diameter of MB.

Fig. S4. Elemental mapping images of MB-CuTA nanoparticles.

Fig. S5. (A) Content of Fe in different volumes of MB-CuTA dispersions. (B) The number of MBs in different volumes of MB-CuTA dispersions.

Fig. S6. XPS spectra of Fe 2p.

Fig. S7. Concentration-dependent ROS production of (A) CuTA nanoparticles and (B) Fe_3O_4 nanoparticles by using TMB as a substrate.

Fig. S8. Evaluations of the *in vitro* cytotoxicity.

Fig. S9. Histopathological examination.

Fig. S10. The therapeutic efficiency of Fe-CuTA.

Fig. S11. (A) General view of biofilm stained by crystal violet in 96-well plate. (B) SEM images of MRSA biofilms in different groups.

Fig. S12. 3D CLSM images of biofilm stained by Hoechst (blue) and coppersensor-1 (red).

Fig. S13. 3D CLSM images of MRSA biofilm stained by stained by Hoechst (blue) and ROS probe (red).

Fig. S14. (A) Correlation analysis between sample. (B) Volcano plots of DEGs in MRSA treated with Fe_3O_4 nanoparticles compared to Saline.

Fig. S15. (A) Volcano plots of DEGs in macrophages treated with Fe_3O_4 nanoparticles compared to Saline.

Fig. S16. Quantitative analysis of (A) pp65, (B) p65, (C) CD86 and (D) Arg1 protein expression.

Fig. S17. Copper inhibitor reverses antibacterial effects against extracellular/intracellular MRSA of Fe-CuTA.

Fig. S18. (A) In situ elemental mapping images of the infected macrophages.

Fig. S19. Photographs of bacterial biofilms around titanium disks in different groups for 3 d.

Fig. S20. SEM images of implants collected from infected tissues in different groups for 3 days.

Fig. S21. (A) FCM analysis of macrophages ($CD11b^+F4/80^+$) infiltration at the infection site and (B) the quantitative analysis.

Fig. S22. Immunofluorescent staining for CD206 across various treatment groups for 3 d.

Fig. S23. (A) Pictures of live MRSA colonies in infected tissues in different groups for 1 d.

Fig. S24. (A) Weight change curves of infected mice after various treatments.

Fig. S25. *In vivo* toxicity evaluation of MB-CuTA.

Fig. S26. Assessment of bacterial dissemination risk *in vivo* and *in vitro*.

Fig. S27. (A) Total and (B) extracellular CFUs in peritoneal lavage fluid determined 16 h after the different treatments.

Fig. S28. (A) FCM analysis of M2-like macrophages ($CD11b^+F4/80^+CD206^+$) and N2-like neutrophils ($CD45^+Ly6G^+CD170^+$) after different treatments in peritoneal lavage fluid.

Fig. S29. (A) Quantification of M2-like macrophages ($CD11b^+F4/80^+CD206^+$) in lymph nodes.

Fig. S30. Quantification of Th1 cells ($CD3^+CD8^-CD4^+IFN\text{-}\gamma^+$) in all spleen lymphocytes after different treatment.

Fig. S31. Levels of IFN-γ in serum.

Table S1 Comparison of therapeutic effects among MB-CuTA and other agents.

Table S2. List of detailed primer sequences for MRSA mRNA.

Table S3. List of detailed primer sequences for macrophage mRNA.

Supplementary Experimental Section

Supplementary Methods

XPS test Purified Fe-CuTA nanoparticles (3 mL, 2.5 mg Cu/mL) were centrifuged at 12000 rpm for 15 min to discard the supernatant. Then, 3 mL acetic acid buffer (pH 4.5 or 7.4) was added to disperse Fe-CuTA nanoparticles. After incubation at 200 rpm/min and 37 ℃ for 0.5h, the samples were taken out to freeze-drying and prepare the sample for the X-ray photoelectron spectroscopy test (Thermo ESCALAB).

Cell culture The RAW264.7 macrophage cell line (ATCC TIB-71) was maintained in Dulbecco's Modified Eagle Medium (DMEM) supplemented with 10% fetal bovine serum (FBS). Bone marrow-derived macrophages (BMDMs) were generated from C57BL/6 mice using established differentiation protocols. Specifically, bone marrow cells were aseptically flushed from femurs and tibias, then cultured in RPMI 1640 medium containing 10% FBS, 1% penicillin-streptomycin (P/S), and 20 ng/mL macrophage colony-stimulating factor (M-CSF). The culture medium was replenished on days 3 and 5 post-isolation to support cellular differentiation. Fully differentiated BMDMs were harvested on day 7 for subsequent experimental applications.

Bacteria and biofilm culture The methicillin-resistant Staphylococcus aureus strain (MRSA, ATCC 43300) was utilized in this study. A single bacterial colony was inoculated into 5 mL of Lysogeny Broth (LB) and incubated at 37°C with 200 rpm orbital shaking for 12-16 hours. Bacterial cells were collected by centrifugation at 10000 rpm for 3 minutes followed by three sequential washes with sterile physiological saline (0.9% NaCl). For biofilm cultivation, 200 μL aliquots of bacterial suspension (10^7 CFU/mL) in LB medium supplemented with 1% (w/v) glucose were aseptically transferred to 96-well plates. Biofilm formation was achieved through static incubation at 37°C for 48 hours. Finally, the LB medium and planktonic bacteria were removed and the biofilm attached to the bottom of 96-well plates was harvested.

Cell viability study The cytotoxic effects of Fe-CuTA nanoparticles were evaluated using the Cell Counting Kit-8 (CCK-8, Beyotime, China) assay according to manufacturer's protocol. Raw 264.7 cells were seeded in 96-well culture plates and exposed to serially diluted concentrations of Fe-CuTA nanoparticles with 0-200 μg/mL CuTA under standard culture conditions (37°C, 5% CO_2) for 24 hours. Following treatment, 10% (v/v) CCK-8 reagent in DMEM (100 μL/well) was introduced to each well. Plates were subsequently incubated for 90 minutes in light-protected

conditions. Absorbance measurements were performed using a spectrophotometric microplate reader with dual-wavelength detection (450 nm test wavelength, 630 nm reference wavelength). Relative cell viability was calculated using the formula: Cell viability (%) = $(OD_{treated}-OD_{control})/OD_{control} \times 100\%$. RAW264.7 cells were seeded in a 12-well plate at a density of 2×10^5 cells per well and incubated overnight under standard culture conditions (37°C, 5% CO_2). Following treatment with various concentrations of Fe-CuTA nanoparticles for 24 hours, a live/dead cell viability assay was conducted using a staining kit (Beyotime, China). Cells were incubated with Calcein-AM/PI working solutions for 30 minutes, after which the culture medium was removed and cells were washed with PBS. Finally, the slides were examined under CLMS (Nikon, Japan). To investigate the hemocompatibility of Fe-CuTA nanoparticles, a hemolysis assay was conducted using fresh blood samples from healthy mice. Briefly, 1.5 mL of whole blood was collected. Red blood cells (RBCs) were isolated by centrifugation at 1000 rpm for 10 minutes and washed three times with PBS. The purified RBCs were then resuspended in PBS to achieve a final concentration of 2%. Subsequently, 1 mL of the RBC suspension was incubated with varying concentrations of Fe-CuTA nanoparticles for 4 hours at 37°C. As controls, PBS and deionized water (ddH_2O) were similarly mixed with the RBC suspension to serve as negative and positive controls, respectively. After incubation, all samples were centrifuged at 1000 rpm for 5 minutes, and the absorbance of the supernatant containing released hemoglobin was measured at 540 nm. The hemolysis ratio was calculated using the following equation: Hemolysis ratio (%) = (ODtreated−ODnegative)/ (ODpositive−ODnegative) × 100%

Coppersensor-1 detection MRSA biofilms were added with Saline, Fe-CuTA nanoparticles or MB-CuTA with or without US stimulation for 4 hours. After washing with cold PBS three times to remove the unbonded materials, biofilms were incubated with 5μM Coppersensor-1 (MCE, USA) and Hoechst (Beyotime, China) for 20 min. Afterwards, the biofilms were immediately imaged using a CLSM (Nikon, Japan).

BCA assay for protein leakage The protein leakage from MRSA was quantified using the PierceTM BCA Protein Assay Kit (Thermo, USA). Specifically, 10^8 CFU/mL of MRSA were incubated with Saline, Fe_3O_4 nanoparticles, and Fe-CuTA nanoparticles (Fe_3O_4 nanoparticles: 0.25 mg/mL; CuTA: 0.025 mg/mL) for 4 hours at 37° C in a controlled environment. Each treatment group was prepared in triplicate. Following incubation, samples were centrifuged at 10000 rpm for 5 minutes at 4° C in a refrigerated centrifuge. The supernatant was carefully collected, and

relative protein leakage was measured using the BCA assay on a microplate reader at an optical density of 562 nm.

Bacterial cell membrane experiment using DiSC$_3$(5) The DiSC$_3$(5) (MaoKang, China) was used to determine the dissipated membrane potential of MRSA after different treatments, after which the fluorescence intensity was recorded using Subsequently, the data obtained from the enzyme marker were analyzed according to the kit instructions..

Mitochondrial respiratory chain activity Cell Mitochondrial Complex I (NADH-CoQ Reductase) Activity Assay Kit and Cell Mitochondrial Complex II Activity Assay Kit (Elabscience, China) were used for respiratory chain activity detection. First, the bacterial samples were cultured in different groups for 4 hours. Subsequently, the data obtained from the microplate reader were analyzed according to the kit instructions.

MDA detection The MRSA samples were cultured in different groups for 4 hours. Following bacterial cell lysis, the lysate was centrifuged at 10000 × g for 10 minutes. The resultant supernatant was collected and subjected to MDA content analysis using a commercial MDA assay kit (Beyotime, China) according to the manufacturer's instructions.

RT-qPCR For RT-qPCR, total RNA was isolated from MRSA or BMDMs using a commercial RNA extraction kit (Vazyme, China), followed by reverse transcription into complementary DNA (cDNA) with PrimeScript RT reagent. The resulting cDNA was amplified and analyzed with ChamQ Universal SYBR qPCR master mix (Vazyme, Nanjing, China) by ViiA^{TM} 7 Software. Gene expression levels were normalized to those of Gapdh or 16s. The mouse primers and bacterial primers were ordered from GeneScript (Nanjing, China). Detailed primer sequences are provided in Supplementary Table 1 and Supplementary Table 2.

Western bolt analysis For the western bolt analysis, BMDMs cells were incubated overnight in 12-well plates at an initial density of 5×10^5 and treated with culture media supplemented with Saline (control), Fe_3O_4 or Fe-CuTA (Fe_3O_4 NPs: 0.25 mg/mL; CuTA: 0.025 mg/mL) for 8 hours. Cells were then lysed with RIPA lysis buffer on ice for 30min. Protein was collected from the supernatant by centrifuging at 10000 rpm at 4 °C for 10min in a microcentrifuge. Protein samples were denatured in 5× SDS loading buffer, electrophoresed on 4-20% SDS-PAGE, and subsequently transferred onto PVDF membranes. Membranes were blocked with 5% (w/v) bovine serum albumin (BSA) in TBST for 1 h at room temperature, followed by sequential incubation with primary antibodies (1:1000) and HRP-conjugated secondary antibodies (1:10000) according

to standardized protocols. Protein bands corresponding to p65, pp65, CD86, and Arg-1 were visualized using enhanced chemiluminescence detection system (Tanon, China). β-actin immunoreactivity served as the internal control for normalization.

Supplementary Figures

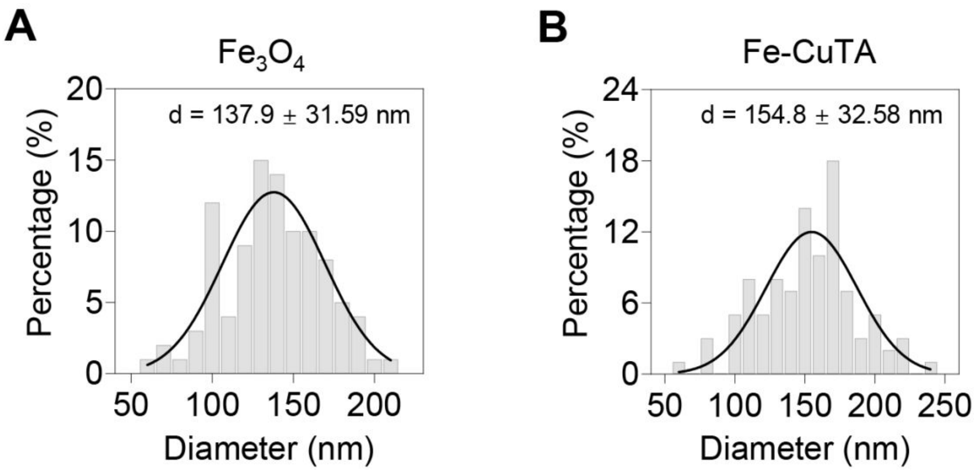

Fig. S1. The diameter distribution of nanoparticles. (**A**) The diameter distribution of Fe_3O_4 nanoparticles (n = 100). (**B**) The diameter distribution of Fe-CuTA nanoparticles (n = 100).

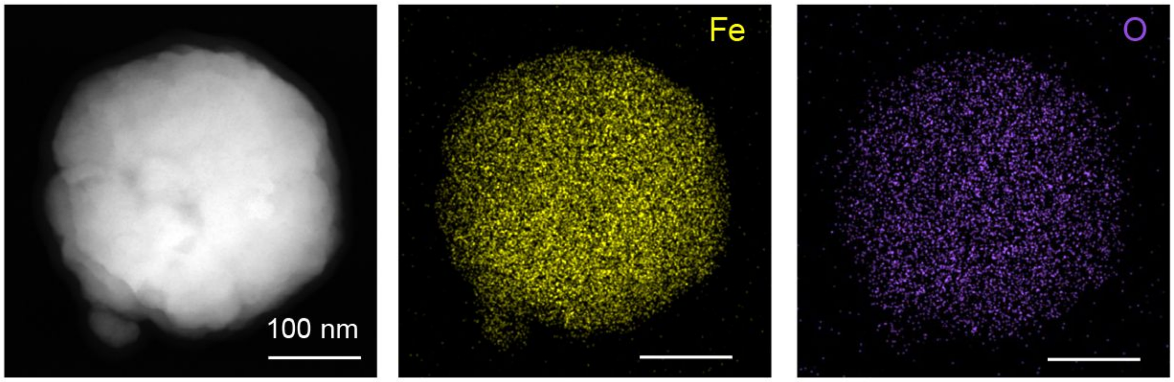

Fig. S2. Elemental mapping of Fe_3O_4 nanoparticle. Scale bar is 100 nm.

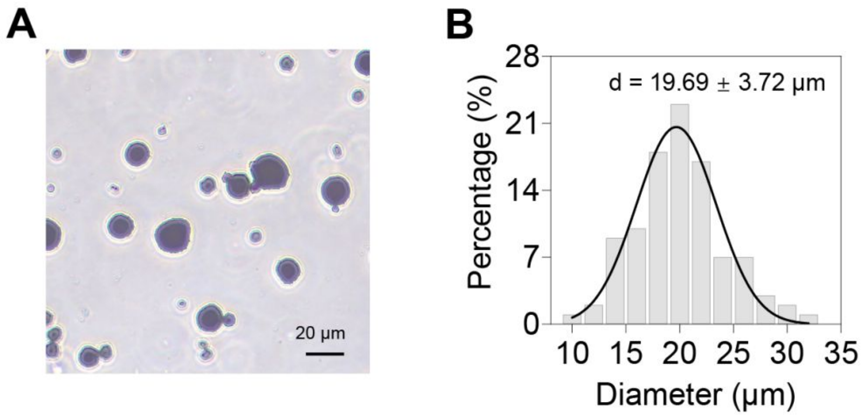

Fig. S3. The diameter of MB. (**A**) Representative bright-field microscopy image of MB. Scale bar is 20 μm. (**B**) The diameter distribution of MB (n = 100).

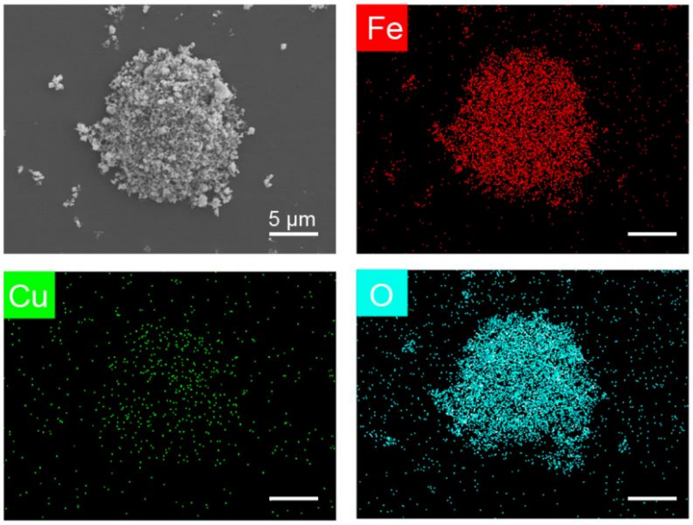

Fig. S4. Elemental mapping images of MB-CuTA nanoparticles. Scale bar of is 5 μm.

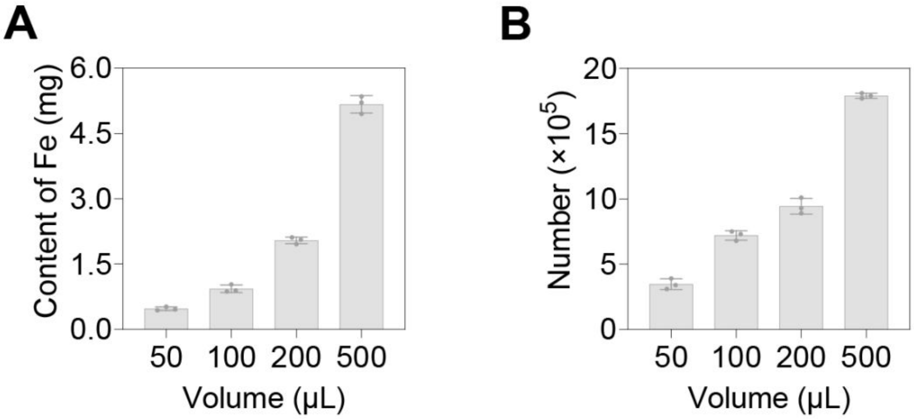

Fig. S5. (**A**) Content of Fe in different volumes of MB-CuTA dispersions. (**B**) The number of MBs in different volumes of MB-CuTA dispersions. Data denote mean ± s.d. (n = 3 independent samples).

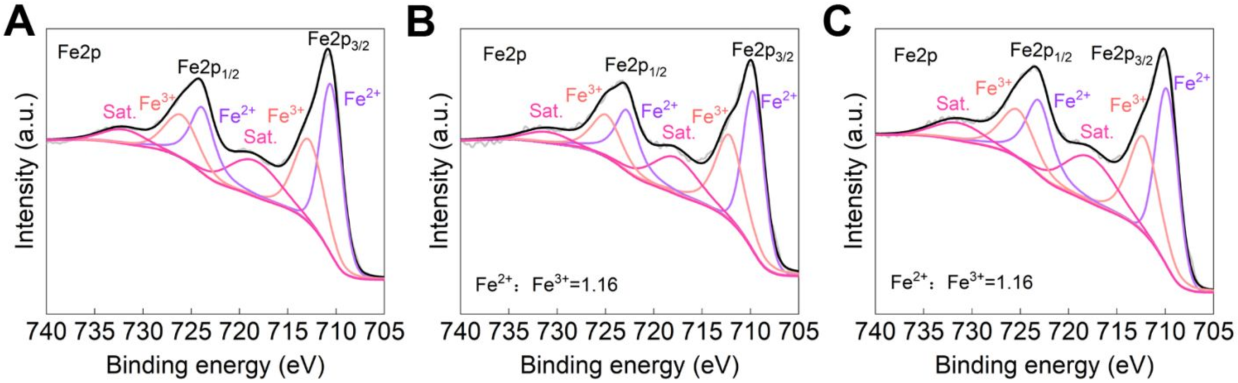

Fig. S6. XPS spectra of Fe 2p. (**A**) XPS spectra of Fe_3O_4 nanoparticles and (**B**) Fe-CuTA nanoparticles in acetic acid buffer (pH = 7.4) for 15 min. (**C**) Fe-CuTA nanoparticles in acetic acid buffer (pH = 4.5) for 15 min.

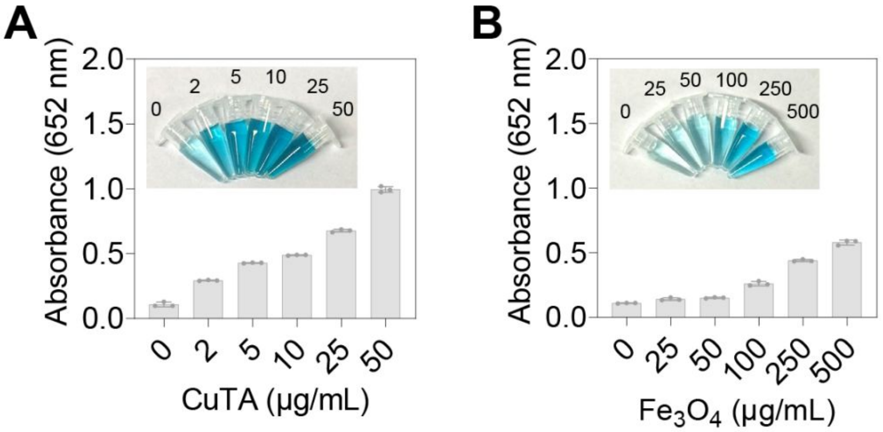

Fig. S7. Concentration-dependent ROS production of (**A**) CuTA nanoparticles and (**B**) Fe_3O_4 nanoparticles by using TMB as a substrate. Inset: Photographs of TMB solutions with different concentration of nanoparticles. Data denote mean ± s.d. (*n* = 3 independent samples).

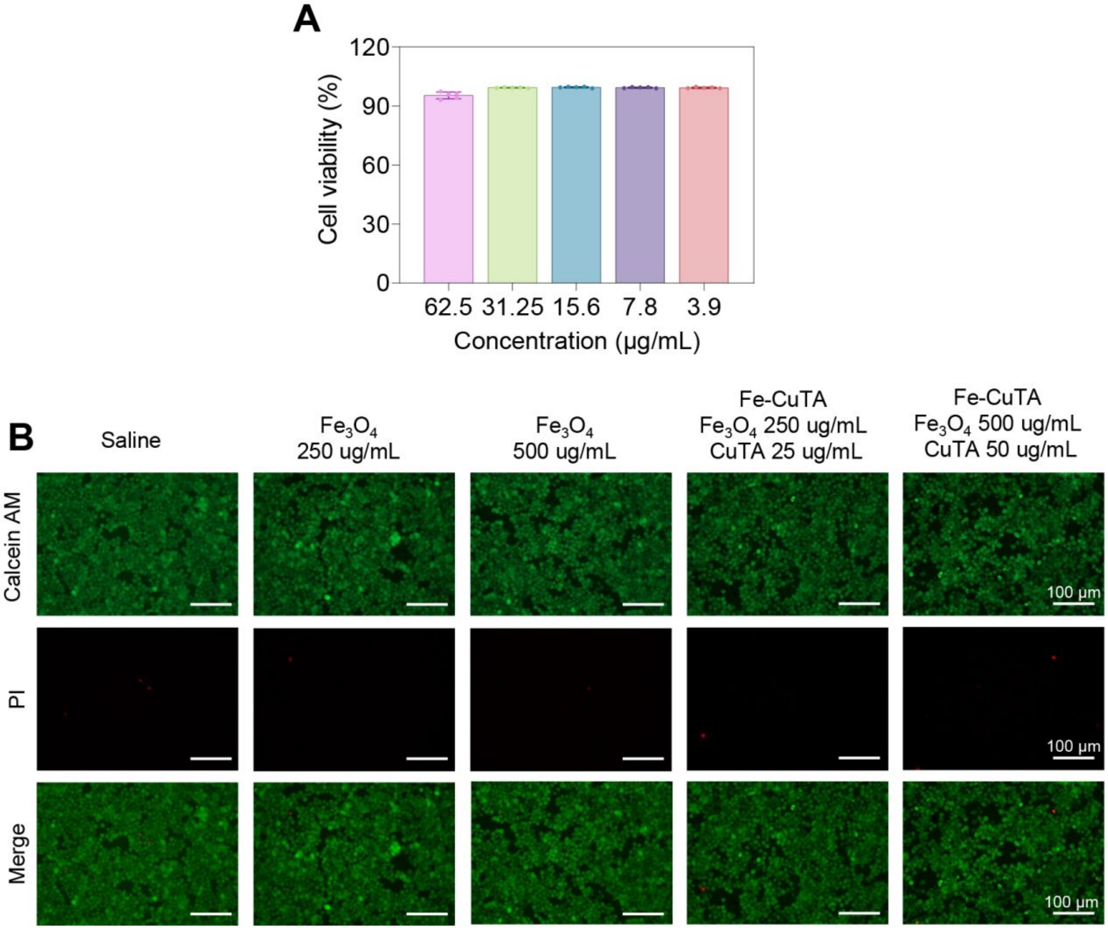

Fig. S8. Evaluations of the *in vitro* cytotoxicity. (**A**) Evaluation of Fe-CuTA biocompatibility for RAW264.7 cells at indicated concentrations (CuTA basis) using the CCK-8 assay. (**B**) Fluorescence microscope images of RAW264.7 cells cultured with varying concentrations of Fe_3O_4 nanoparticles or Fe-CuTA nanoparticles. Scale bar, 100 μm. Data denote mean ± s.d. (*n* =3 independent samples).

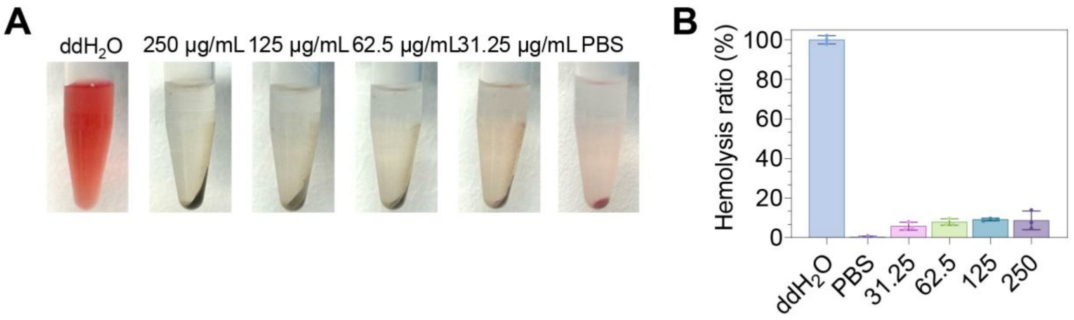

Fig. S9. Histopathological examination. (**A**) Photos of mouse red blood cells (RBCs) incubated with water, PBS, and different concentrations of CuTA for 2 h. (**B**) Hemolysis ratio of different groups. Data denote mean ± s.d. (*n* =3 independent samples).

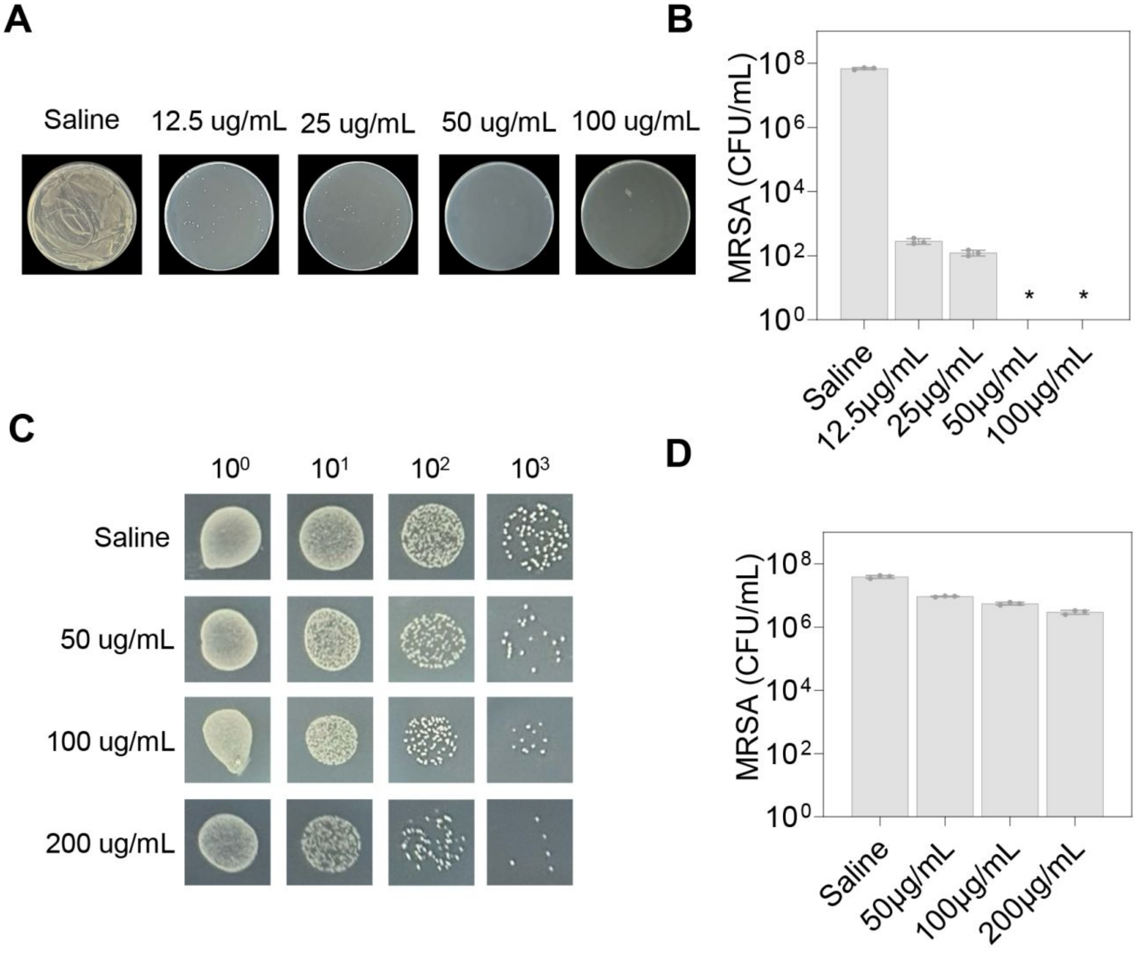

Fig. S10. The therapeutic efficiency of Fe-CuTA. (**A**) Photographs of MRSA colonies cultured in different concentrations of Fe-CuTA groups (CuTA basis) and (**B**) colony-forming units (CFUs) count. (**C**) Photographs of MRSA colonies within biofilms cultured in different concentrations of Fe-CuTA groups (CuTA basis) and (**D**) CFUs count. Data denote mean ± s.d. (*n* = 3 independent bacteria samples).

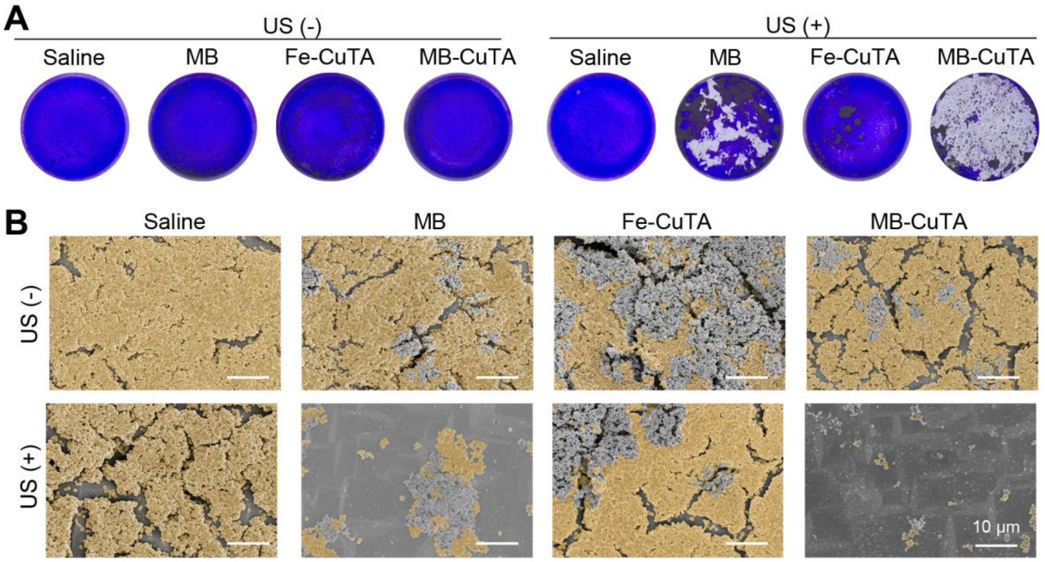

Fig. S11. (**A**) General view of biofilm stained by crystal violet in 96-well plate. (**B**) SEM images of MRSA biofilms in different groups. Scale bar, 10 μm.

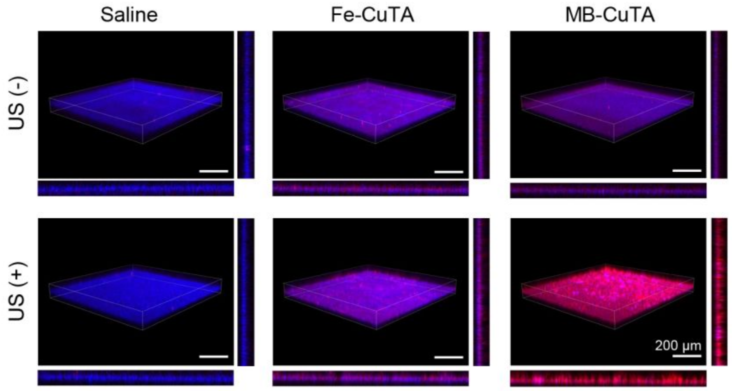

Fig. S12. 3D CLSM images of biofilm stained by Hoechst (blue) and coppersensor-1 (red). Scale bar, 200 μm.

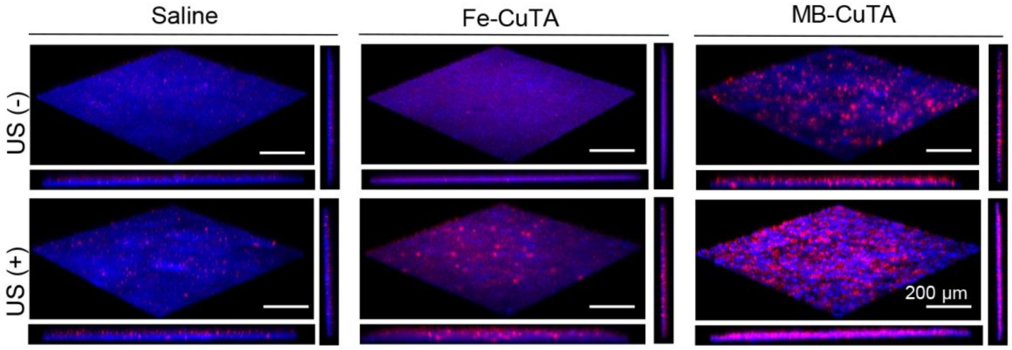

Fig. S13. 3D CLSM images of MRSA biofilm stained by stained by Hoechst (blue) and ROS probe (red). Scale bar, 200 μm.

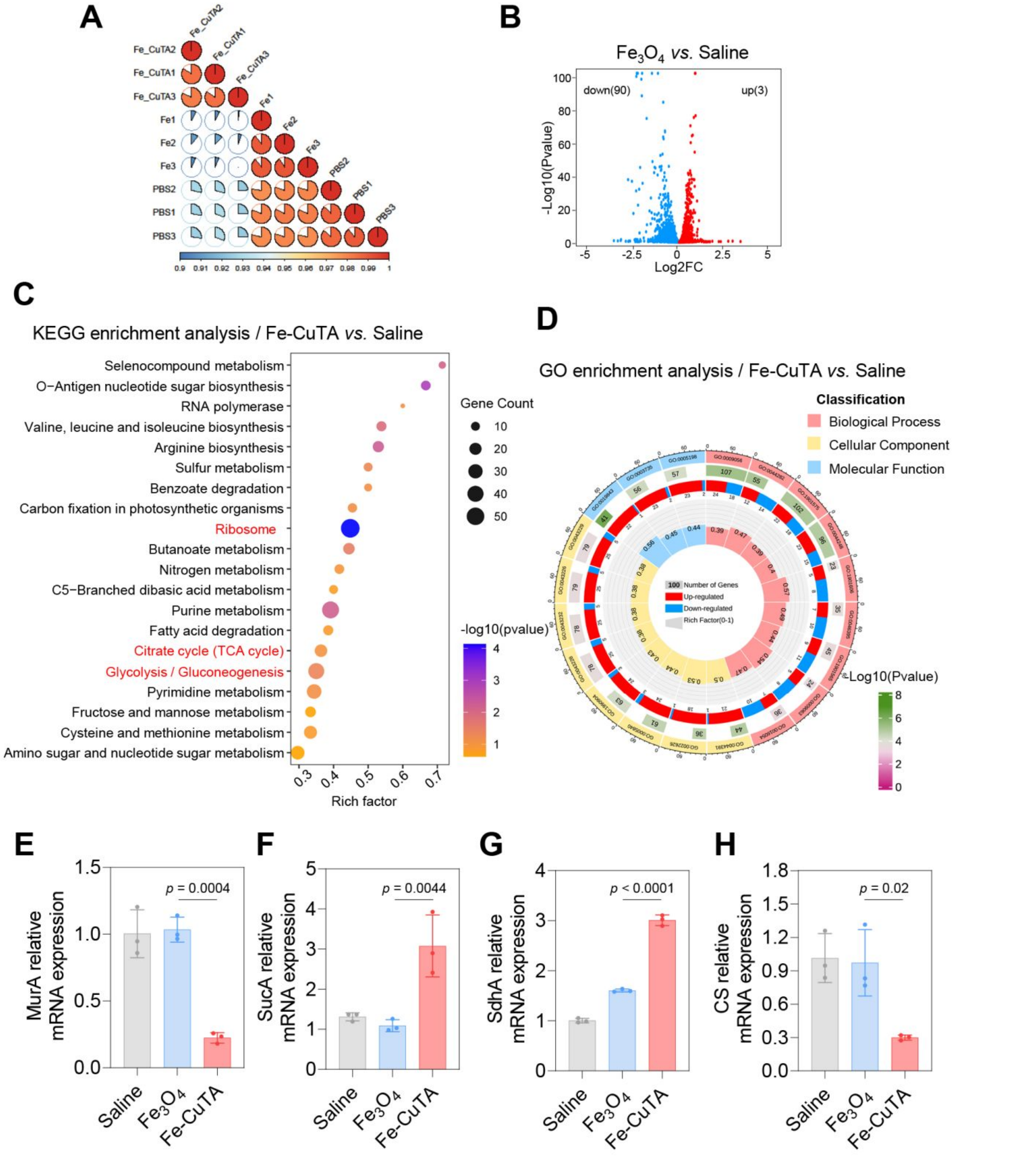

Fig. S14. (**A**) Correlation analysis between sample. (**B**) Volcano plots of DEGs in MRSA treated with Fe_3O_4 nanoparticles compared to Saline. (**C**) KEGG enrichment analysis scatter plot between Fe-CuTA and Saline group. (**D**) GO enrichment analysis between Fe-CuTA and Saline group. (**E** to **H)** qPCR results of typical genes involved in cuproptosis-like pathway. Data denote mean ± s.d. (n = 3 independent samples for RNA sequencing and for **E** to **H**). Significant differences of **E** to **H** were assessed by a two-tailed unpaired Student's t-test.

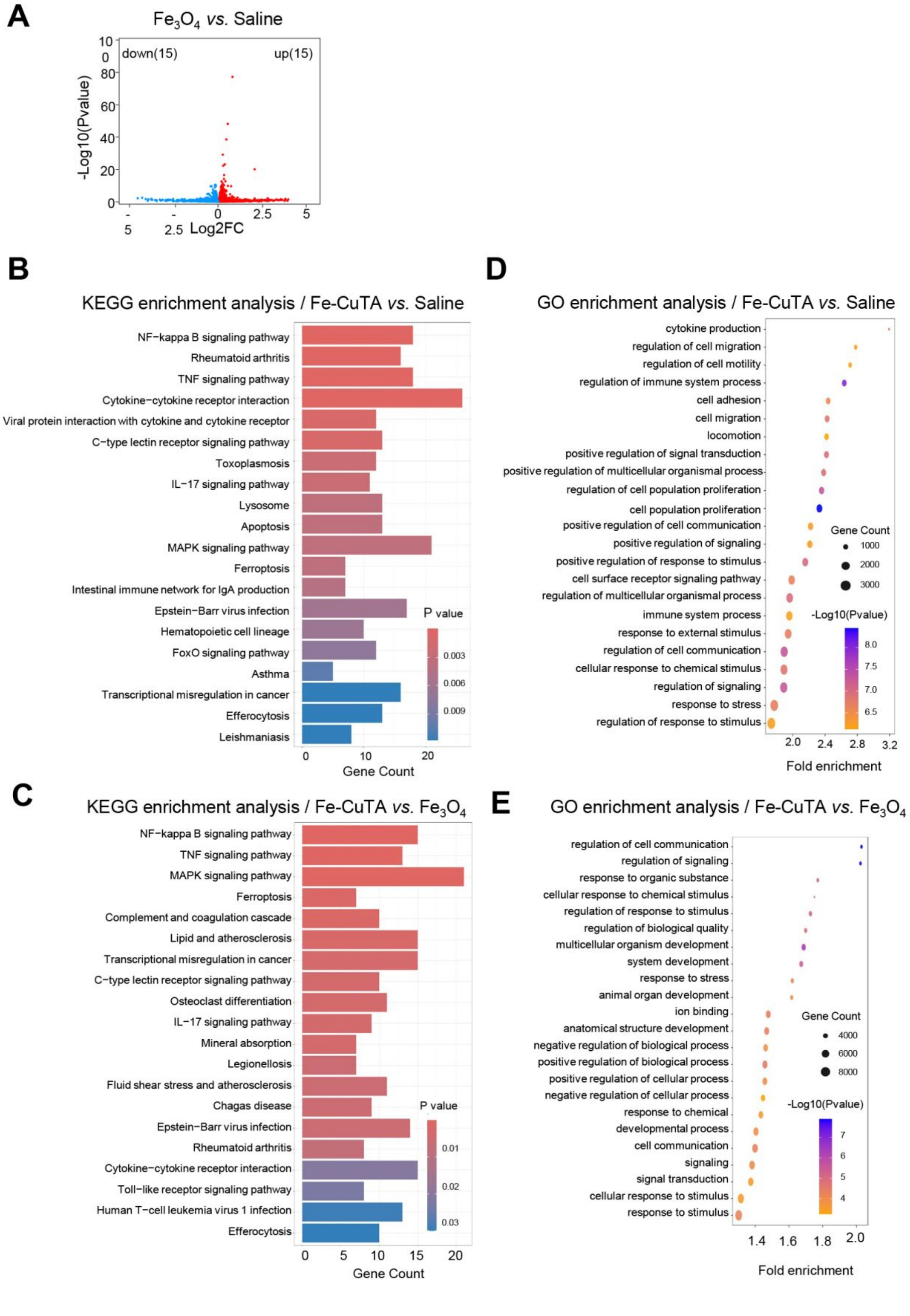

Fig. S15. (**A**) Volcano plots of DEGs in macrophages treated with Fe_3O_4 nanoparticles compared to Saline. (**B** and **C**) KEGG enrichment analysis of macrophages for Fe-CuTA group compared with Saline group and Fe_3O_4 group. (**D** and **E**) GO enrichment analysis of macrophages for Fe-CuTA group compared with Saline group and Fe_3O_4 group. Data denote mean ± s.d. (*n* = 3 independent samples for RNA sequencing)

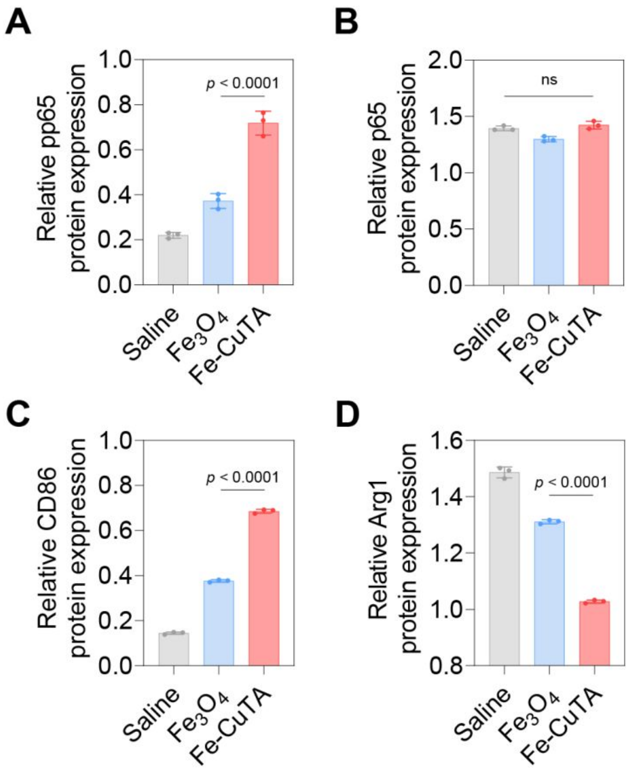

Fig. S16. Quantitative analysis of (**A**) pp65, (**B**) p65, (**C**) CD86 and (**D**) Arg1 protein expression. Data denote mean ± s.d. (n = 3 independent cell samples). Significant differences were assessed by a two-tailed unpaired Student's t-test.

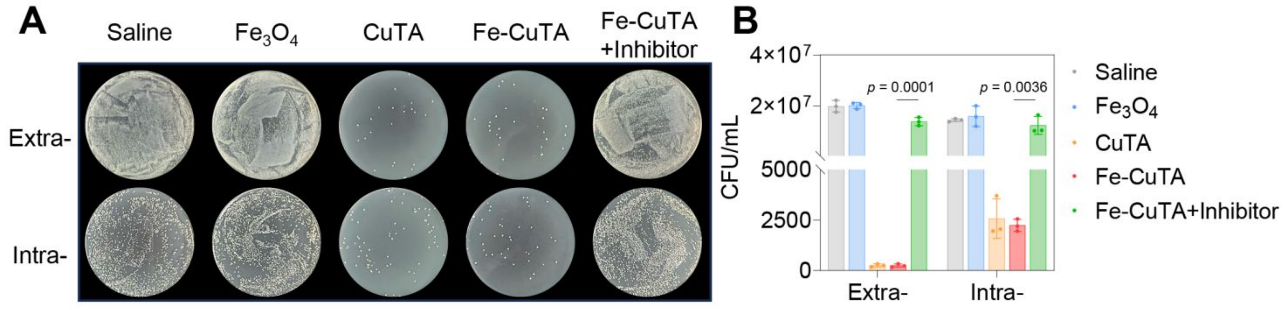

Fig. S17. Copper inhibitor reverses antibacterial effects against extracellular/intracellular MRSA of Fe-CuTA. (**A**) Photos of extracellular and intracellular MRSA colonies with different treatments. (**B**) The number of live extracellular and intracellular MRSA calculated from (A) (n = 3, mean ± s.d.).

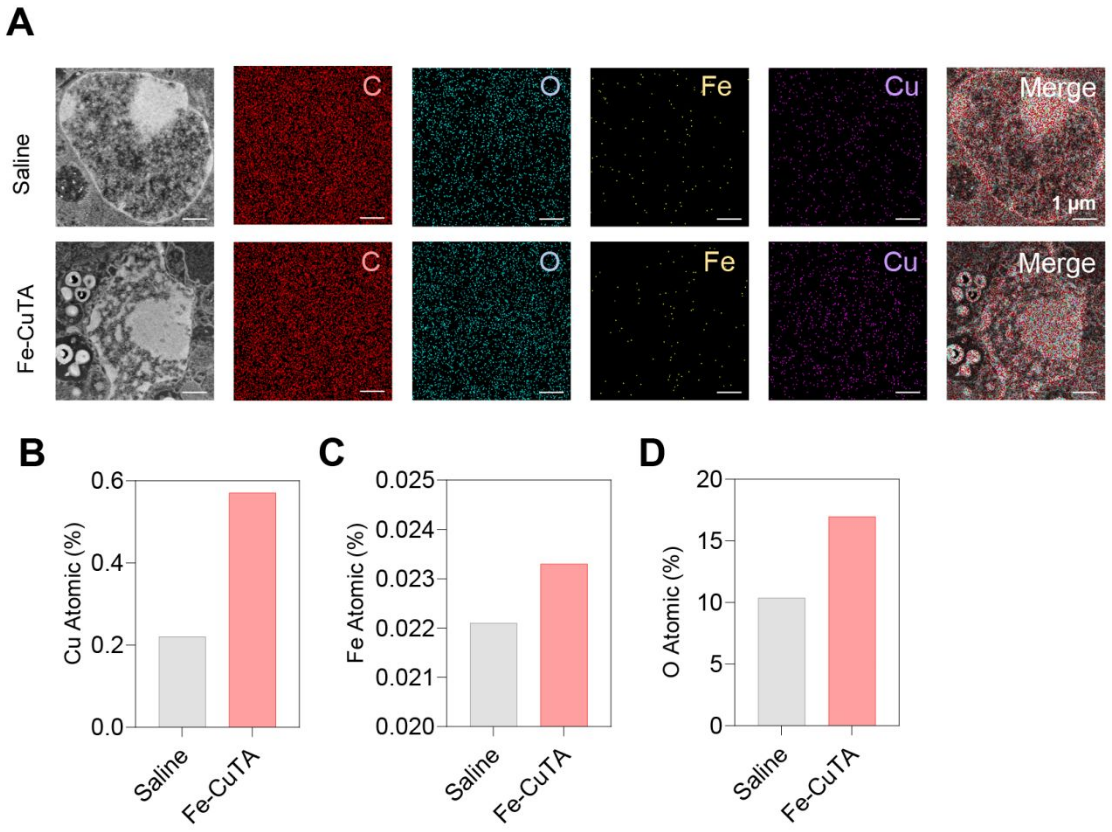

Fig. S18. (**A**) In situ elemental mapping images of the infected macrophages. Atomic percentage of (**B**) Cu, (**C**) Fe and (**D**) O.

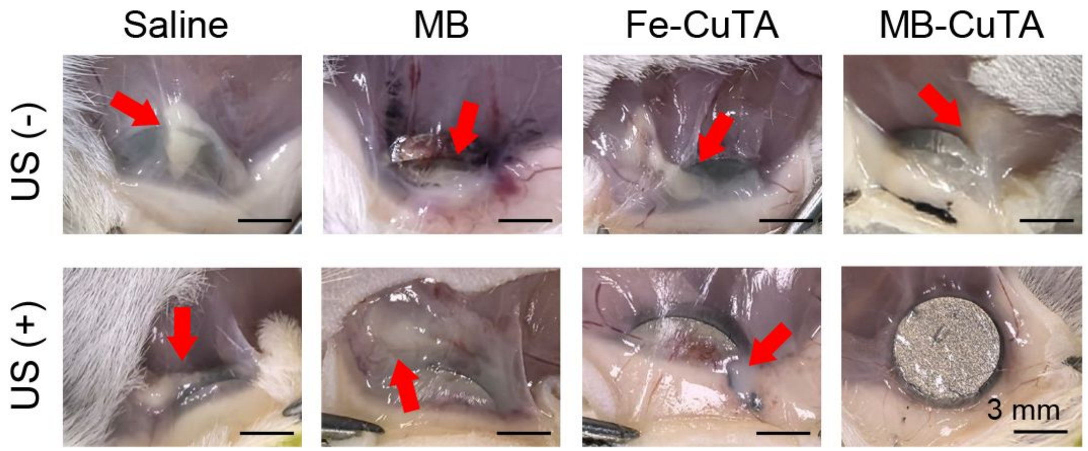

Fig. S19. Photographs of bacterial biofilms around titanium disks in different groups for 3 d. Scale bar, 3 mm.

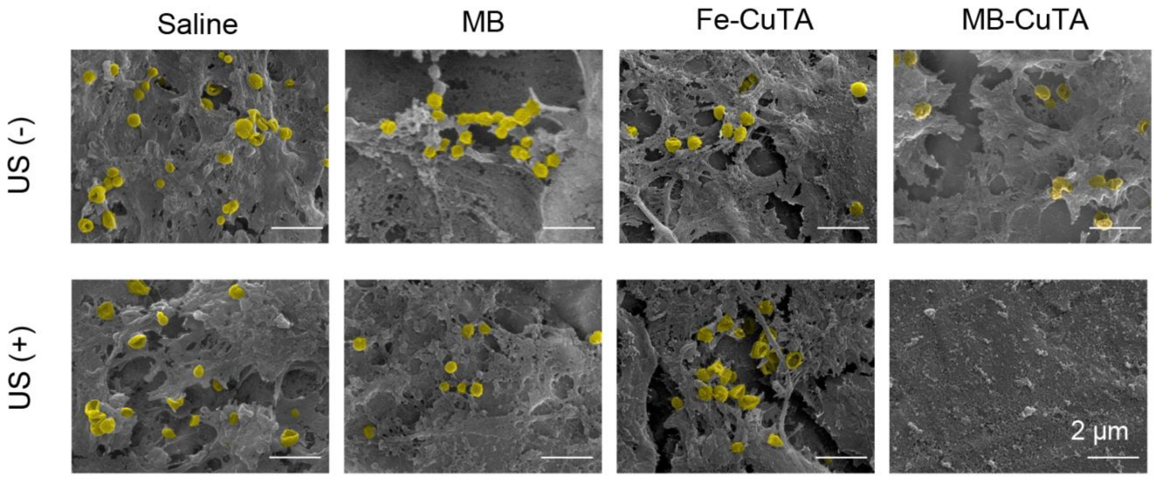

Fig. S20. SEM images of implants collected from infected tissues in different groups for 3 days. The pseudo-colored yellow indicates the location of MRSA. Scale bar, 2 μm.

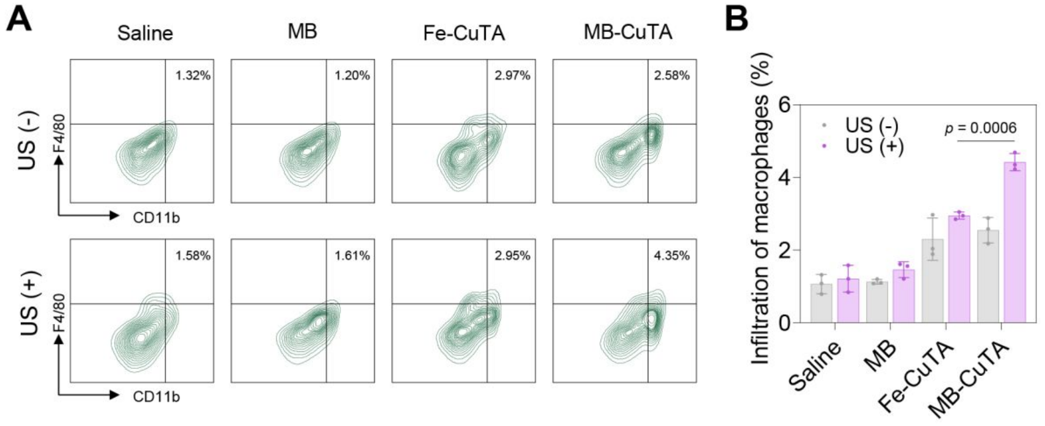

Fig. S21. (**A**) FCM analysis of macrophages ($CD11b^{+}F4/80^{+}$) infiltration at the infection site and (**B**) the quantitative analysis. Data denote mean ± s.d. ($n = 3$ independent samples). Significant differences were assessed by a two-tailed unpaired Student's t-test.

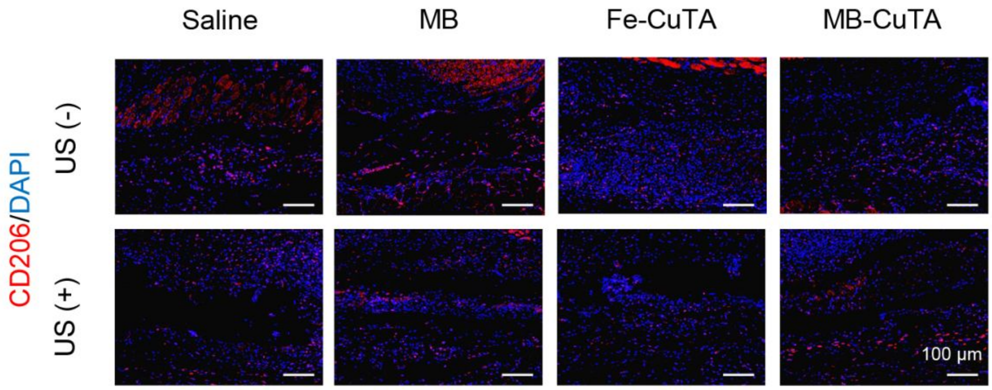

Fig. S22. Immunofluorescent staining for CD206 across various treatment groups for 3 d. Scale bar is 100 μm.

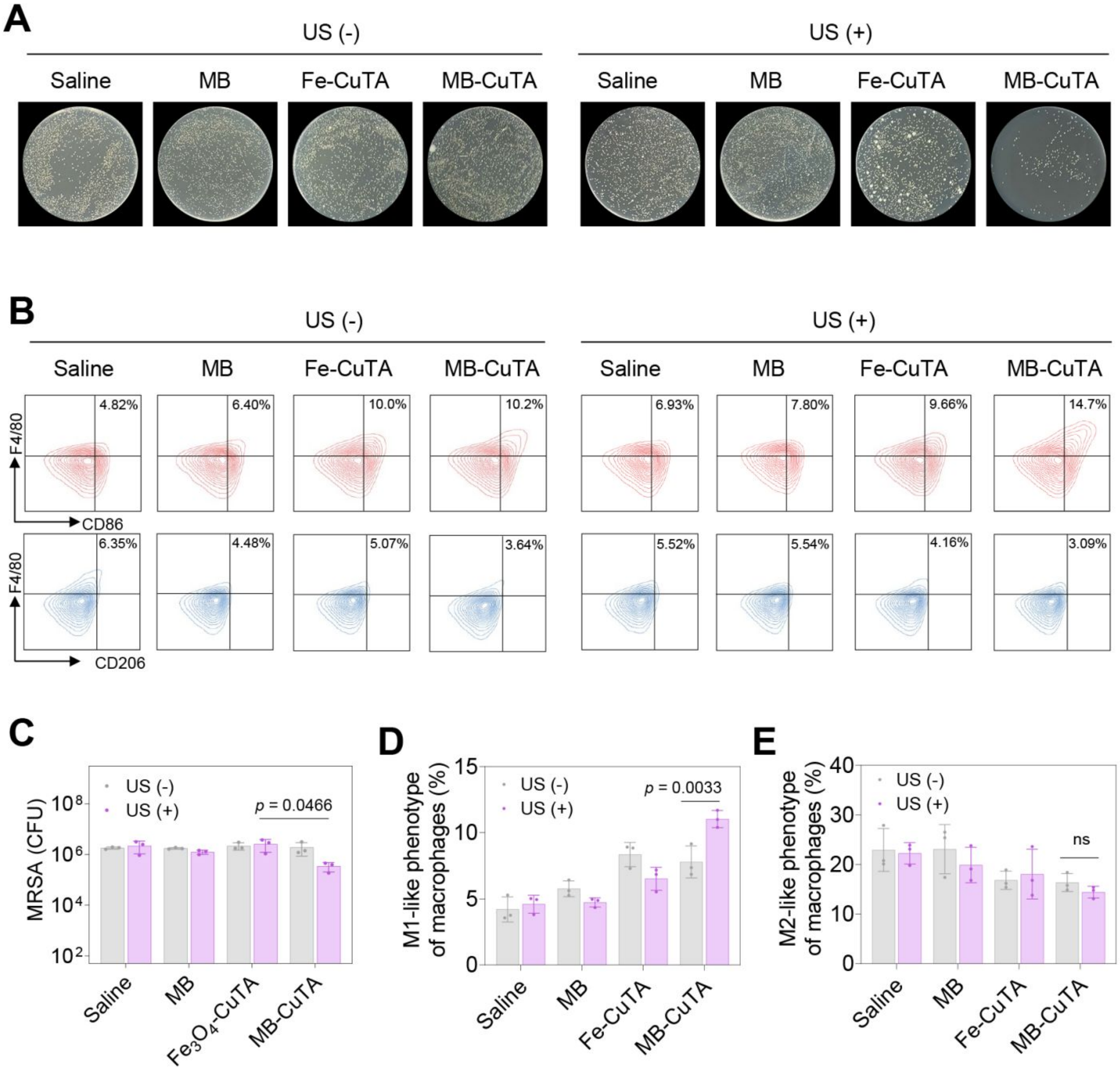

Fig. S23. (**A**) Pictures of live MRSA colonies in infected tissues in different groups for 1 d. (**B**) FCM analysis of M1-like ($CD11b^{+}F4/80^{+}CD86^{+}$) and M2-like ($CD11b^{+}F4/80^{+}CD206^{+}$) macrophages post 1 d of different treatments in the infected tissues. (**C**) Corresponding colony number of live MRSA calculated from (A). (**D**) Quantitative analysis of M1-like phenotype and (**E**) M2-like phenotype macrophages. Data denote mean ± s.d. (n = 3 independent animal samples). Significant differences were assessed by a two-tailed unpaired Student's t-test.

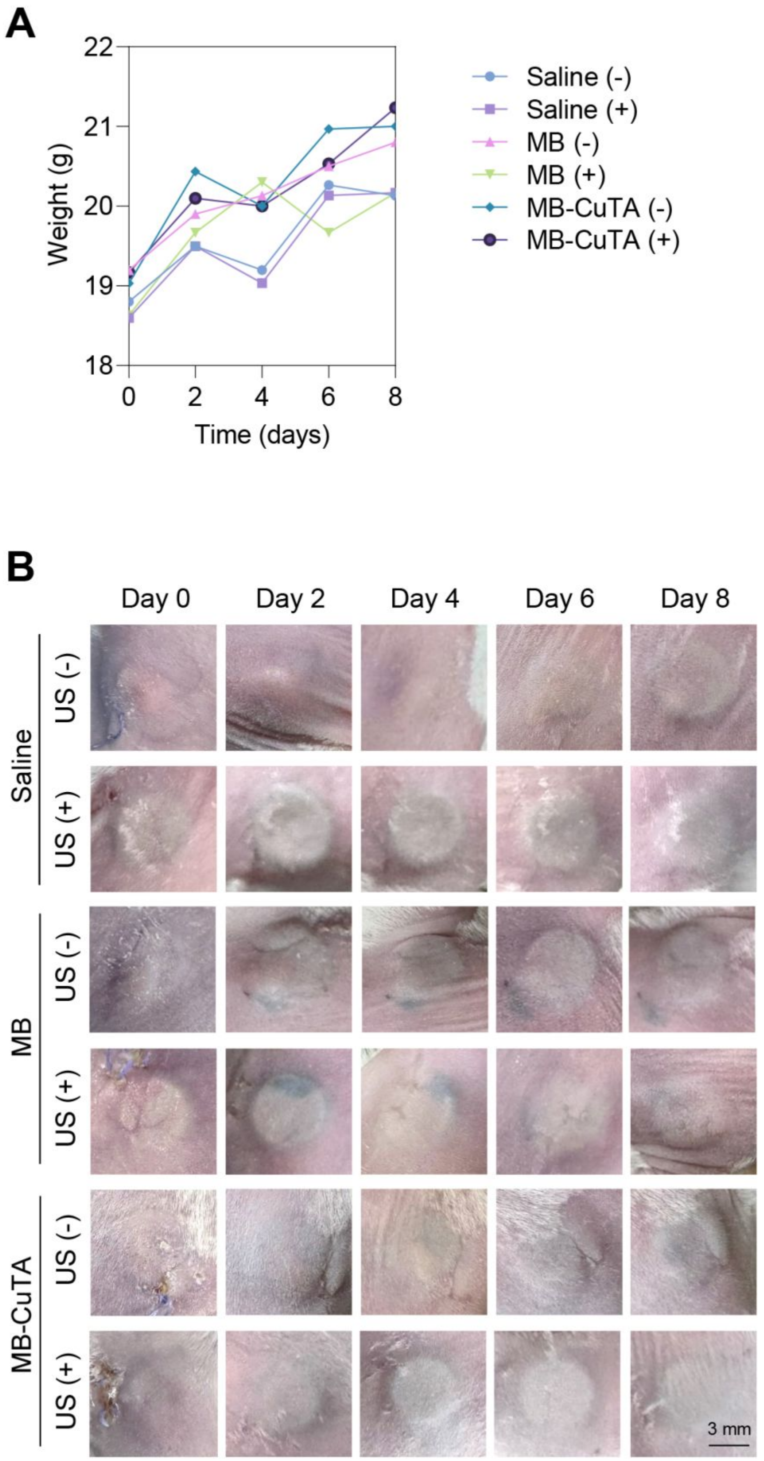

Fig. S24. (**A**) Weight change curves of infected mice after various treatments. Data denote mean ± s.d. (n = 3 independent animal samples). (**B**) Representative photos of implant infection after treatments for 8 d.

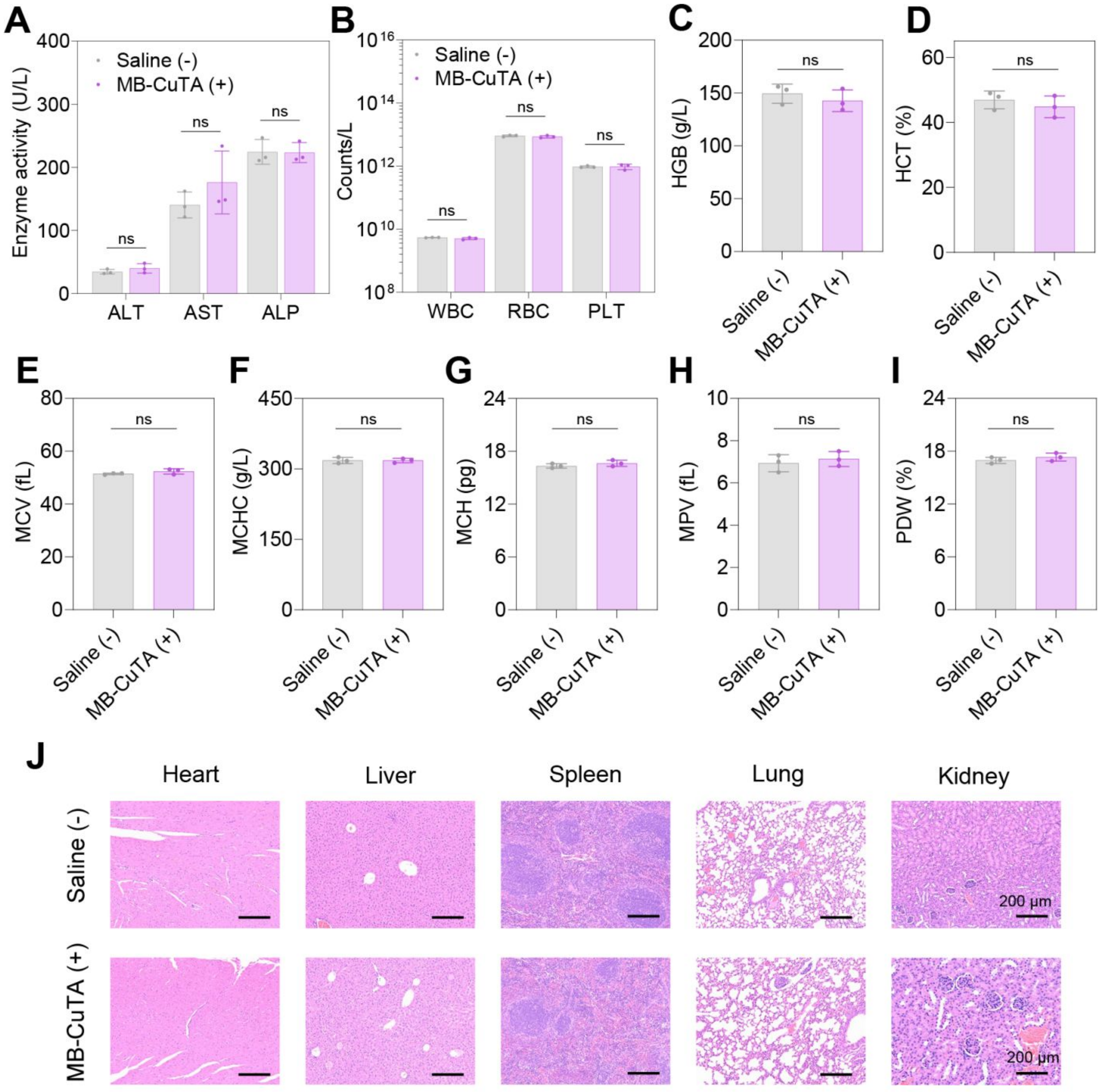

Fig. S25. ***In vivo* toxicity evaluation of MB-CuTA**. (**A**) Alanine aminotransferase (ALT), aspartate aminotransferase (AST), alkaline phosphatase (ALP), (**B**) white blood cells (WBC), red blood cells (RBC), platelets (PLT), (**C**) hemoglobin (HGB), (**D**) hematocrit (HCT), (**E**) mean corpuscular volume (MCV), (**F**) mean corpuscular hemoglobin concentration (MCHC), (**G**) mean corpuscular hemoglobin (MCH), (**H**) mean platelet volume (MPV) and (**I**) platelet distribution width (PDW) levels in the blood at 21d post post-injection of Saline or MB-CuTA under US stimulation (dose of Fe-CuTA = 5mg/kg). (**J**) H&E staining images of major organ sections (heart, liver, spleen, lung, and kidney) from mice post-injection of Saline or MB-CuTA under US stimulation. Scale bar is 200 μm. Data denote mean ± s.d. (*n* = 3 independent animal samples for **A** to **I**). Significant differences were assessed by a two-tailed unpaired Student's t-test. ns: no significant difference.

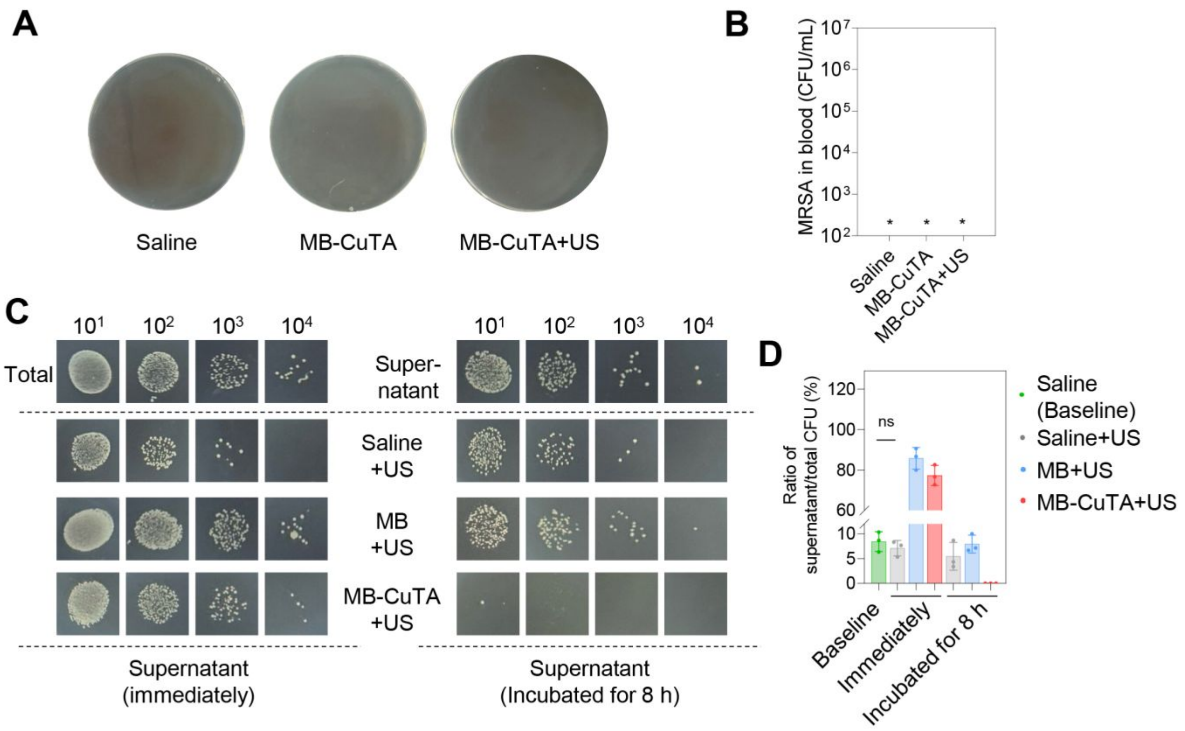

Fig. S26. Assessment of bacterial dissemination risk *in vivo* and *in vitro*. (**A**) Photographs of MRSA colonies plate and (**B**) the number of live MRSA in blood of infected mice after various treatments for 24 h (n = 3, mean ± s.d.), *indicates not detected. (**C**) Photographs of MRSA colonies plate and (**D**) the number of total live MRSA or live MRSA in supernatant of biofilms (n = 3, mean ± s.d.).

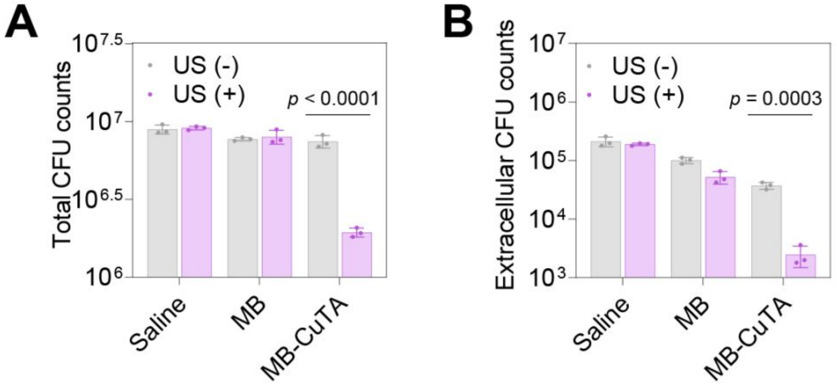

Fig. S27. (**A**) Total and (**B**) extracellular CFUs in peritoneal lavage fluid determined 16 h after the different treatments. Data denote mean ± s.d. (n = 3 independent animal samples). Significant differences were assessed by a two-tailed unpaired Student’s t-test.

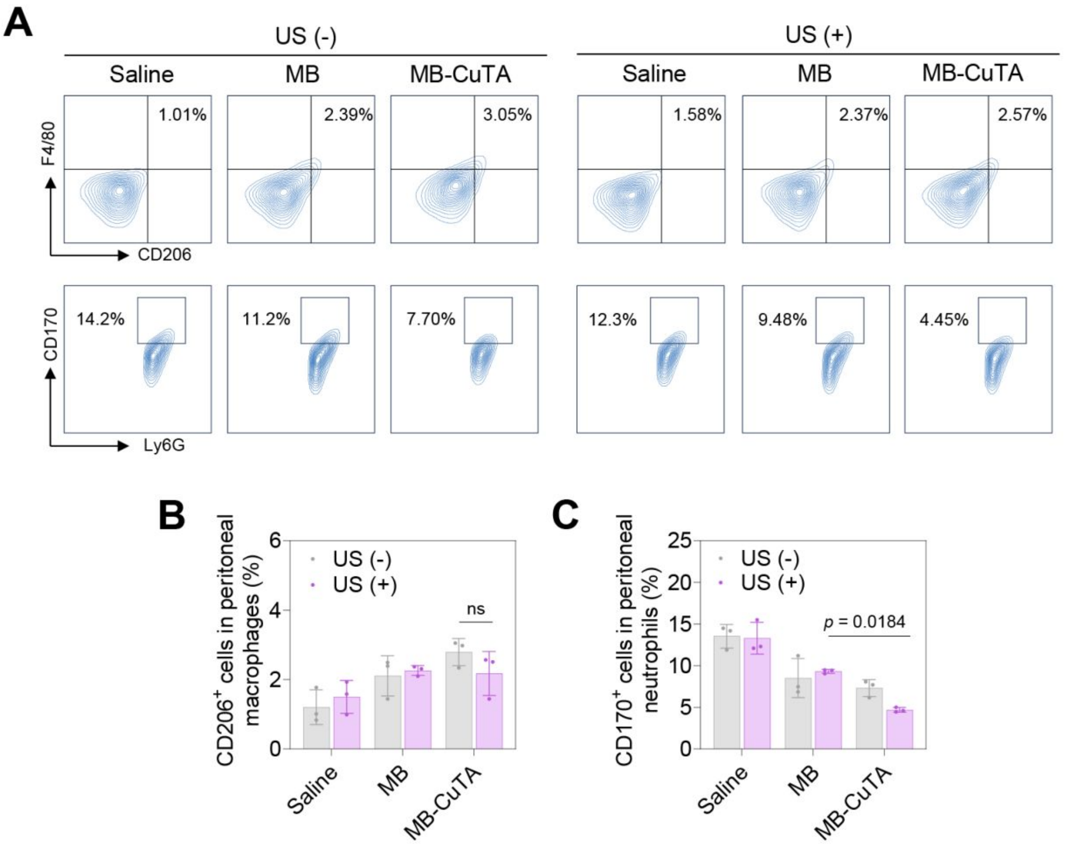

Fig. S28. (**A**) FCM analysis of M2-like macrophages ($CD11b^+F4/80^+CD206^+$) and N2-like neutrophils ($CD45^+Ly6G^+CD170^+$) after different treatments in peritoneal lavage fluid. (**B**) Quantification of M2-like macrophages and (**C**) N2-like neutrophils. Data denote mean ± s.d. (*n* = 3 independent animal samples for **B** and **C**). Significant differences were assessed by a two-tailed unpaired Student's t-test.

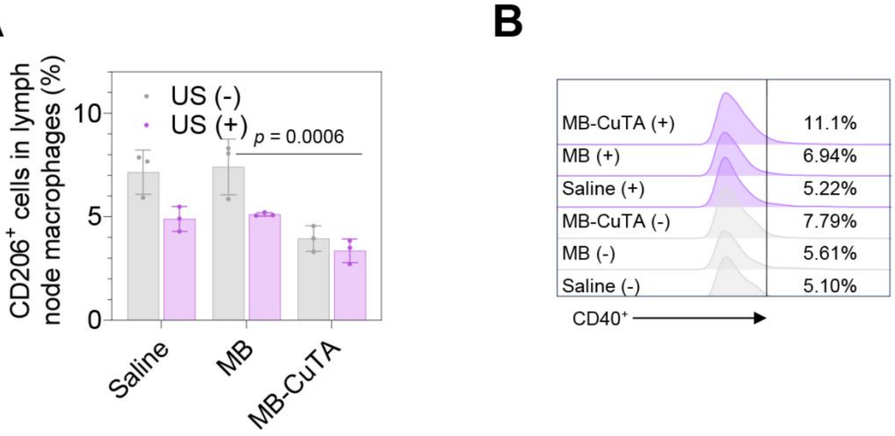

Fig. S29. (**A**) Quantification of M2-like macrophages ($CD11b^+F4/80^+CD206^+$) in lymph nodes. (**B**) FCM analysis of $CD40^+$ DCs in lymph nodes. Data denote mean ± s.d. (*n* = 3 independent animal samples for **A**). Significant differences were assessed by a two-tailed unpaired Student's t-test.

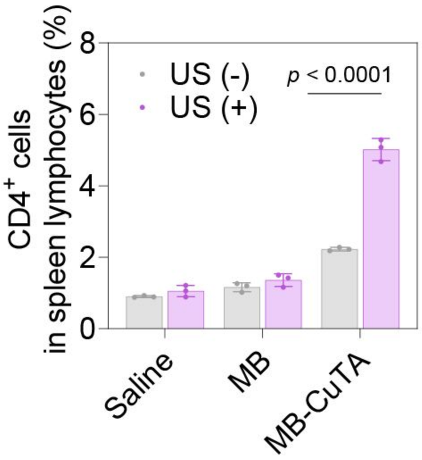

Fig. S30. Quantification of Th1 cells ($CD3^+CD8^-CD4^+IFN\text{-}\gamma^+$) in all spleen lymphocytes after different treatment. Data denote mean ± s.d. ($n = 3$ independent animal samples). Significant differences were assessed by a two-tailed unpaired Student's t-test.

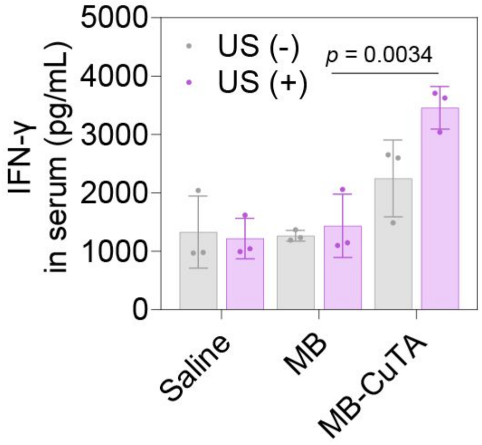

Fig. S31. Levels of IFN-γ in serum. Data denote mean ± s.d. ($n = 3$ independent animal samples). Significant differences were assessed by a two-tailed unpaired Student's t-test.

Supplementary Tables

Table S1. Comparison of therapeutic effects among MB-CuTA and other agents.

Material	Mechanisms for biofilm disruption	Specific parameters	Efficacy of biofilm elimination	Synergistic therapy	Ref.
MB-CuTA	Ultrasonic cavitation	1 MHz, 1.0 W/cm^2, 50%, 10 min (Ultrasound)	99.998% of MRSA biofilms	ROS generation; Toxicity and immune activation of copper ions	This work
UNB	Ultrasonic cavitation	1 MHz, 2.0 W/cm^2, 50%, 5 min (Ultrasound)	99.99% of *P. aeruginosa* biofilms	Sonodynamic therapy	[1]
Ag-TiO_2-LA Janus nanoparticles	NO gas propelling nanomotor	1 W/cm^2, 3 MHz (Ultrasound)	Approximately 99% of *S. aureus* and *E. coli* biofilms	Sonodynamic therapy; Photothermal therapy	[2]
Gold nanoparticles	Laser-induced vapour nanobubbles	1.69 J/cm^2 (Laser)	Approximately 98% of *S. aureus* biofilms	Antibiotics	[3]
PFH/Rif@NDs	Laser-induced vapour nanobubbles	725 nm, 221 mW/cm^2, 20 Hz, 60 s (Laser)	Over 98% of *S. aureus* biofilms	Antibiotics	[4]
MNPs@Ag@HA	Magnetic attraction penetration	2000 Gauss (Magnetic field)	95% of *S. aureus* biofilms	Antibiotics; ROS generation	[5]
Vanc.DNase@ MoS_2/Ag-Fe_3O_4	DNase mediated perforation; Magnetic attraction penetration	50-500 mT, 5 min (Magnetic field)	96% to100% of *E. faecalis* and *S. mutans* biofilms	Antibiotics	[6]
AuAgCu@DNase hydrogels	DNase mediated perforation	/	51.2 % of MRSA biofilms and 64.6 % of *E. coli* biofilms	Photothermal therapy	[7]
CSNP-DNase-GOX	DNase mediated perforation; Electrostatic interactions	/	85.63 % of dual-species biofilms (*S. enterica* and *L. monocytogenes*)	ROS generation	[8]
Dex-K_nE_m and Dex-G_nE_m	Electrostatic interactions	/	Over 90% of *P. aeruginosa* biofilms	/	[9]
Ir@Co_3O_4 (S)	Spiky morphology	/	Over 90% of MRSA biofilms	ROS generation	[10]

Table S2. List of detailed primer sequences for MRSA mRNA.

Gene	Primer (Forward)	Primer (Reverse)
SgtB	CGGTCATTGAAGCAAACAACGTC	CTGCTCCCTCAATCGTAT
CopB	GTGGAAGTCAGAGCAGGCGAAAG	TACTAGCCGTTCAAGTGCGAAATCG
CopZ	TCACAAGAAATTTTAAATGTTGAAGG	ACGACATCGTAACCTTGATCTTC
PckA	GCTGGTACAGAGCGTGGTGT	TCCCACCAGTCCATCCAGTGT
SdhA	AAGACGCTGGTCGTGAAG	CAGCAAAGATTGGCGTGA
SdhB	GATGACAATCAGCAGCGTG	CACGAATCACTGGTTTTGTAGG
SucA	AGCAGCACAAGATGATACACAAC	ACCACCCGTAGAATAGCCTTTC
SucB	ACGACAATACTCCACAACAAAATG	ACTTACTTCAGCAAGATTCACACC
MurA	TCGCGGTGATATCTTTGTGCGT	GTTGGGAATCCTGGGTGTGGT
CS	AAAGCATTTTACGGCAAAGAAGT	TCGTTGTGATTTTGGAGATTGAA
16s	CCATAAAGTTGTTCTCAGTT	CATGTCGATCTACGATTACT

Table S3. List of detailed primer sequences for macrophage mRNA.

Gene	Primer (Forward)	Primer (Reverse)
Cd80	ACCCCCAACATAACTGAGTCT	TTCCAACCAAGAGAAGCGAGG
Il6	TAGTCCTTCCTACCCCAATTTCC	TTGGTCCTTAGCCACTCCTTC
Cxcl2	CCAACCACCAGGCTACAGG	GCGTCACACTCAAGCTCTG
Il1β	GCAACTGTTCCTGAACTCAACT	ATCTTTTGGGGTCCGTCAACT
Tnfα	CCCTCACACTCAGATCATCTTCT	GCTACGACGTGGGCTACAG
Il12	GGTCTCAACCCCCAGCTAGT	GCCGATGATCTCTCTCAAGTGAT
Cd206	CTCTGTTCAGCTATTGGACGC	CGGAATTTCTGGGATTCAGCTTC
Arg1	CTCCAAGCCAAAGTCCTTAGAG	AGGAGCTGTCATTAGGGACATC
Gapdh	AGGTCGGTGTGAACGGATTTG	TGTAGACCATGTAGTTGAGGTCA

REFERENCES

[1]. Ma, J.; Teng, Z.; Ding, L.; Peng, X.; Xie, Y.; Long, Q.; Jiang, L.; Huo, S.; Liang, X.; Liu, G., Ultrasound-triggered nano-bomb: Bacteria-targeted oxygen-supplying liposomes as seed bubbles for enhanced mechano-sonodynamic therapy of pseudomonas aeruginosa biofilms. *Adv. Funct. Mater.* **2025**, 2501347.

[2]. Zhao, W.; Ding, Q.; Zhou, B.; Liu, J.; Shi, Y.; Liu, C.; Li, C.; Dong, B.; Qi, M.; Kim, J. S.; Wang, L., Nitric oxide-actuated titanium dioxide janus nanoparticles for enhanced multimodal disruption of infectious biofilms. *Adv. Funct. Mater.* **2024,** *34* (45), 2407626.

[3]. Teirlinck, E.; Xiong, R.; Brans, T.; Forier, K.; Fraire, J.; Van Acker, H.; Matthijs, N.; De Rycke, R.; De Smedt, S. C.; Coenye, T.; Braeckmans, K., Laser-induced vapour nanobubbles improve drug diffusion and efficiency in bacterial biofilms. *Nat. Commun.* **2018,** *9* (1), 4518.

[4]. Cao, B.; Lyu, X.; Wang, C.; Lu, S.; Xing, D.; Hu, X., Rational collaborative ablation of bacterial biofilms ignited by physical cavitation and concurrent deep antibiotic release. *Biomaterials* **2020,** *262*, 120341.

[5]. Wang, X.; Wu, J.; Li, P.; Wang, L.; Zhou, J.; Zhang, G.; Li, X.; Hu, B.; Xing, X., Microenvironment-responsive magnetic nanocomposites based on silver nanoparticles/gentamicin for enhanced biofilm disruption by magnetic field. *ACS Appl. Mater. Interfaces* **2018,** *10* (41), 34905-34915.

[6]. Baig, M. M. F. A.; Fatima, A.; Gao, X.; Farid, A.; Ajmal Khan, M.; Zia, A. W.; Wu, H., Disrupting biofilm and eradicating bacteria by Ag-Fe_3O_4@MoS_2 MMPs nanocomposite carrying enzyme and antibiotics. *J. Control. Release* **2022,** *352*, 98-120.

[7]. Lin, N.; Wang, M.; Gong, H.; Li, N.; Liu, F.; Wu, Y.; Sun, X.; Yang, Q.; Tan, X., Immobilizing dnase in ternary auagcu hydrogels to accelerate biofilm disruption for synergistically enhanced therapy of mrsa infections. *Int. J. Biol. Macromol.* **2024,** *277*, 134518.

[8]. Lin, Q.; Sheng, M.; Kang, Z.; Xu, J.; Gao, Y.; Ma, S.; Xin, B.; Tan, Y., Synergistic and antibiofilm activity of DNase I and glucose oxidase loaded chitosan nanoparticles against dual-species biofilms of listeria monocytogenes and salmonella. *Int. J. Biol. Macromol.* **2024,** *269*, 131943.

[9]. Yu, D.; He, J.; Zhang, X.; Liu, Y.; Yang, Y.; Yin, L.; Luan, S.; Tang, H., Biofilm penetrating and disrupting polymers to effectively treat endotracheal-tube-associated biofilm infections. *Acta Biomater.* **2025,** S1742-7061(25), 00473-8.

[10]. Xiao, S.; Xie, L.; Gao, Y.; Wang, M.; Geng, W.; Wu, X.; Rodriguez, R. D.; Cheng, L.; Qiu, L.; Cheng, C., Artificial phages with biocatalytic spikes for synergistically eradicating antibiotic-resistant biofilms. *Adv. Mater.* **2024,** *36* (32), 2404411.
